# The effectiveness of two different exercise approaches in adolescent idiopathic scoliosis: A single-blind, randomized-controlled trial

**DOI:** 10.1371/journal.pone.0249492

**Published:** 2021-04-15

**Authors:** Hikmet Kocaman, Nilgün Bek, Mehmet Hanifi Kaya, Buket Büyükturan, Mehmet Yetiş, Öznur Büyükturan

**Affiliations:** 1 Department of Physiotherapy and Rehabilitation, Prosthetics-Orthotics Physiotherapy, Karamanoglu Mehmetbey University, Karaman, Turkey; 2 Department of Physiotherapy and Rehabilitation, Faculty of Health Sciences, Lokman Hekim University, Ankara, Turkey; 3 Faculty of Medicine, Ahi Evran University, Kırşehir, Turkey; 4 School of Physical Therapy and Rehabilitation, Ahi Evran University, Kırşehir, Turkey; 5 Department of Orthopedics and Traumatology, Faculty of Medicine, Ahi Evran University, Kırşehir, Turkey; Prince Sattam Bin Abdulaziz University, College of Applied Medical Sciences, SAUDI ARABIA

## Abstract

**Objectives:**

The purpose of this study was to compare the efficacy of two different types of exercise methods in patients with adolescent idiopathic scoliosis.

**Methods:**

In total, 28 subjects with adolescent idiopathic scoliosis with a mild curve magnitude (10°–26°) were randomly divided into two groups: the Schroth group (n = 14) and the core group (n = 14). The patients in the Schroth group were treated with supervised Schroth exercises, and the patients in the core group were treated with supervised core stabilization exercises; both groups performed the exercises for three days per week for a total of 10 weeks, and both were given additional traditional exercises to perform. Assessment included Cobb angle (Radiography), trunk rotation (Adam’s test), cosmetic trunk deformity (Walter Reed Visual Assessment Scale), spinal mobility (Spinal Mouse), peripheral muscle strength (Biodex System 4-Pro), and quality of life (Scoliosis Research Society-22 questionnaire).

**Results:**

It was found that patients in the Schroth group showed greater improvement in Cobb angles, thoracic trunk rotation angle, cosmetic trunk deformity, spinal mobility, and quality of life than those in the core group (p<0.05), except for in lumbar trunk rotation angle. Peripheral muscle strength improvement was greater in the core group than in the Schroth group (p<0.05).

**Conclusion:**

Schroth exercises are more effective than core stabilization exercises in the correction of scoliosis and related problems in mild adolescent idiopathic scoliosis, and core stabilization exercises are more effective than Schroth exercises in the improvement of peripheral muscle strength.

**Trial registration:**

NCT04421157

## Introduction

Adolescent idiopathic scoliosis (AIS) is a three-dimensional deformity of the spine with an unknown etiology; it is clinically described as a curvature of the spine in the coronal plane greater than 10 degrees. It presents in 2–2.5% of adolescents and is often accompanied by a rotation of the spine in the axial plan and an alteration in sagittal physiological curvature [1]. AIS affects body alignment, the spinal column, and soft tissue structures, leading to physical problems such as postural abnormality, cosmetic trunk deformity, degraded flexibility of the spinal column, change in the characteristics of the erector spine muscle, back pain, and, in severe cases, reduced respiratory function [2]. Various treatment approaches have been proposed to deal with these complications and others [3, 4].

Depending on the severity of the curve, treatment approaches consist of exercises, bracing, and surgery to prevent, correct, or halt the progression of the deformity caused by AIS [4]. A review of the literature revealed that exercises are often recommended to decrease progression, correct postural behavior, increase neuro-motor control of the spine, and to improve spine and thoracic flexibility, muscle strength, and elasticity [5]. Exercise is almost always a part of the treatment plan for patients with AIS. In mild cases, exercise may be the main treatment, and it may serve as an adjunct in more severe cases [3].

There are several approaches to exercise designed to treat scoliosis, including the Schroth method [6]. The Schroth method is a scoliosis-specific exercise approach commonly performed in scoliosis rehabilitation that uses postural, scoliosis-specific sensorimotor and breathing exercises [6, 7]. The treatment program consists of scoliotic posture correction with the help of exteroceptive and proprioceptive stimulations and mirrors, isometrics, and other exercises to lengthen or strengthen the asymmetrical muscles while maintaining a specific breathing pattern. Auto-correction is a basic component of the Schroth method, described as the patient’s ability to decrease the spinal deformity using active postural realignment of the spine in three dimensions. The Schroth method is intended to improve the patient’s motor control of their posture through the repetition of corrective movements with progressively less feedback [6, 8]. In several studies, the Schroth method was demonstrated to improve Cobb angles, slow curve progression, reduce the need for surgery, increase back muscle strength, and boost breathing function [7, 9, 10]. However, although the Schroth method is widely used in AIS rehabilitation, there are few randomized controlled studies on the effectiveness of Schroth exercises [7].

Other recently introduced stabilizing physiotherapeutic exercises used in the conservative treatment of AIS include yoga, Pilates, and core stabilization (CS). These exercises focus on core strength training and spinal stability [11–13]. CS exercises are commonly used for improving neuromuscular control, strength, and the endurance of different muscles around the spine to correct and maintain the alignment of the spine [14]. One of the major therapeutic aims of CS exercises used in the treatment of scoliosis is to improve spinal stability [13]. Previously, limited studies have demonstrated that CS exercises are effective in the treatment of scoliosis and in the improvement of posture in patients with AIS [13, 15, 16]. For example, Gür et al. [15] found CS exercises to be more effective than traditional exercises (breathing exercises, posture training, spinal flexibility exercises, stretching exercises) for the reduction of pain and the correction of vertebral rotation in patients with moderate AIS. Other studies have concluded that CS exercises decrease Cobb angle, increase lumbar muscle strength, and improve sitting balance in patients with AIS [13, 16].

The Scientific Society on Scoliosis Orthopaedic Rehabilitation and Treatment (SOSORT) has stated that evidence on the effectiveness of conservative treatment in AIS is scarce and that the isolated effects of therapeutic and corrective exercises on AIS patients have not been clearly defined [4]. Recently, a systematic review identified the positive effects of therapeutic exercises based on the Schroth method or CS exercises in the management of AIS. The review also suggested that further studies of better methodological quality are required to confirm the effectiveness of such exercises and to determine the best therapeutic exercise intervention for AIS [17]. To the best of our knowledge, no research has previously been done comparing the effects of the Schroth method with those of CS exercises in patients with AIS. Thus, there is a need for randomized controlled studies on the effectiveness of different methods of exercise in the treatment of AIS. This study was done to compare the effects of Schroth exercises and CS exercises on Cobb angle, spinal mobility, trunk rotation, peripheral muscle strength, cosmetic deformity, and health-related quality of life in patients with AIS.

## Materials and methods

### Study design

The study design was a randomized, single-blind 1:1 parallel-group study and was conducted at Kırşehir Ahi Evran University Faculty of Medicine, Departments of Orthopedics and Traumatology and the School of Physical Therapy and Rehabilitation between October 2019 and March 2020. The study proposal was approved by the Research Ethics Board of Kırşehir Ahi Evran University Faculty of Medicine (08/10/2019). Prior to the study, written and oral consent was given by all participants and their families. The study was conducted in accordance with the Declaration of Helsinki principles. The authors confirm that all ongoing and related trials for this study were registered. Due to an error of omission, the trial was registered retrospectively on May 12, 2020, before the data was analyzed (ClinicalTrials.gov Identifier: NCT04421157). We hereby state that all future trials will be registered prospectively.

### Participants

Participants in this study were adolescents diagnosed with AIS by a physician according to Lenke criteria and referred to receive an exercise treatment at the School of Physical Therapy and Rehabilitation of Kırşehir Ahi Evran University. The inclusion criteria were as follows: diagnosed with AIS, aged between 10–18 years, Lenke curve type 1 [18], Risser stage ≤ 3 [19], and a Cobb angle of 10–30°. Participants with a history of neuromuscular, cardiovascular, pulmonary, vestibular, or rheumatological diseases were excluded. Patients were also excluded if they had been prescribed brace treatment, had non-idiopathic scoliosis, had taken medicine periodically, had received any previous surgical or conservative treatment of the spine, were unable to participate, or were reluctant to receive treatment.

### Interventions

Supervised exercise treatment programs were performed 90 minutes three times per week for 10 weeks with patients in both groups. The Schroth group (SG) performed Schroth exercises, and the core group (CG) performed CS exercises.

Both groups also performed supervised traditional exercises. These traditional exercises consisted of stretching exercises (particularly for the muscles on the concave side of the curve), posture training, breathing exercises, and spinal flexibility exercises. The exercise program gradually increased in intensity in accordance with each participant’s functional improvement.

The CS training principles, demonstrated in previous studies [13, 14, 20]. The CS training consisted of three phases. The primary purpose of the first phase was to activate the core muscles to improve proprioception and muscular coordination in spinal areas. Thus, the training program focused on local muscle stability training (transversus abdominis, diaphragm, and multifidus) in static positions. In the second and third phases, exercises were made more intense to improve muscular stability and endurance [20].

The CS exercises were explained and assisted by a certified and experienced physiotherapist (B.B.). In the CG, participants were taught isolated activation of the transversus abdominis in the first session while performing an abdominal drawing-in maneuver. Once isolated activation of the transversus abdominis was achieved, the participants were asked to perform the maneuver during all stability exercises and in different positions (quadruped, sitting, and standing). The first and second phases each lasted three weeks, and the third phase took place over four weeks. Each training session began with 10 minutes of warm-up exercises and finished with 10 minutes of cool-down exercises; both warm-up and cool-down exercises included breathing and stretching exercises. The number of repetitions was adjusted according to the participant’s exercise tolerance. During the first week of each phase, the number of repetitions of each exercise was 7–10, and this progressed to 10–15 based on the patient’s physical tolerance. Training progression was achieved by adding movements to the extremities, working in different positions, the use of TheraBands, the use of exercise balls, and by using the weight of the patient’s body’. The rules and improvements of CS exercises are shown in S1 Appendix.

Schroth exercises consist of passive and active postural auto-correction exercises done repeatedly and based on kinesthetic and sensorimotor principles. The ultimate goal of the Schroth method is to enable the patient to consciously maintain correct posture in their daily living activities [8]. To achieve this requires repeating corrective movements performed to improve postural motor control. Schroth exercises also include strength and endurance training of postural muscles in order to improve the curve, raise the patient’s self-image, and reduce pain [6]. In this study, the Schroth exercises progressed from more to less passive support, from more to less feedback, and from lying to sitting or standing positions, according to the patient’s ability to perform the specific exercise [6, 9].

The Schroth exercises were demonstrated and supervised by a certified and experienced physiotherapist (H.K.). Patients were placed in an asymmetric position to maximize correction in trunk symmetry. The Schroth program includes exercises for rotational breathing, spinal elongation, de-flexion, stretching, de-rotation, and strengthening, and these exercises were performed to improve the curvature, muscle strength, and endurance of postural muscles. During the Schroth exercises, rice bags, foam blocks, a stool, and long sticks were used to adjust the posture and give passive support. The intensity of the Schroth exercises was gradually increased depending on the patient’s improvement in exercise performance by decreasing the amount or degree of passive support, changing the patient’s position, and adjusting the sets and repetitions of exercises. The rules and improvements of Schroth exercises are shown in S2 Appendix.

### Outcome measurements

Socio-demographic data (age, gender, weight, height, body mass index) were collected in an in-person interview. Cobb angle, trunk rotation angle, spinal mobility, cosmetic trunk deformity, peripheral muscle strength, and quality of life of the participants were assessed by the same researcher who performed the interview.

#### Primary outcome

*Cobb angle*. Curve magnitude was evaluated using the Cobb method, which is considered the gold standard for monitoring scoliosis progression. Cobb angles, in degrees, were obtained using a standard anterior-posterior standing full spine radiograph [21].

#### Secondary outcomes

*Trunk rotation angle*. Trunk rotation angle (ATR) was evaluated using Bunnell’s scoliometer and Adam’s forward bend test. The patients were asked to bend forward, and the angle of trunk rotation (the angle between the horizontal plane and a plane across the posterior aspect of the trunk) was measured using the apical vertebrae of the curve. In order to have clinical significance, the change in ATR must be >4°. This measurement has been proven to be sensitive, specific, and reliable [22].

*Cosmetic trunk deformity*. The cosmetic trunk deformity was evaluated using the Walter Reed Visual Assessment Scale (WRVAS). The WRVAS was designed to assess the perceived physical deformity of patients with idiopathic scoliosis. The test allows patients to describe their perception of their deformity. The WRVAS demonstrates seven visible aspects of spinal deformity, including shoulder level, body curve, head pelvis, flank prominence, rib prominence, scapular rotation, and head rib pelvis. Scores for each catagory range from 1 (no deformity) to 5 (the worst deformity), and the total score is generated from the sum of the scores from the seven domains [23]. The WRVAS was found to have high reliability and validity for AIS patients to assess their perception of deformity [24].

*Spinal mobility*. Spinal mobility was evaluated using a skin-surface computer-aided device (The Spinal Mouse^®^ System, Idiag, Fehraltorf, Switzerland). Spinal Mouse is a non-invasive external measurement method developed to evaluate segmental and global thoracal and lumbar mobility on multiple planes [25]. It has been found to be a practical, valid, and reliable device for the clinical assessment of AIS patients [26]. In this study, measurements were taken from the spinous process of C7 to the top of the anal crease (roughly S3). Maximal flexion and maximal extension were measured in the sagittal plane (SP), and maximal right and left lateral flexion positions were measured in the frontal plane (FP). Total SP and FP movements were recorded [27].

*SRS-22 questionnaire*. In this study, the Scoliosis Research Society-22 (SRS-22) questionnaire was used to assess health-related quality of life. The SRS-22 questionnaire is a valid self-reported instrument for the assessment of quality of life related to scoliosis. It includes five domains: self-image, function, pain, mental health (five questions each), and satisfaction with treatment (two questions). The questionnaire has a total of 22 items that are scored from 1 (worst) to 5 (best) for each item. The final score is the average of these five domains. This instrument has been found to have good validity and test-retest reliability [28].

*Muscle strength*. A Biodex System 4-Pro dynamometer (Biodex, Inc., Shirley, New York) was used to assess patients’ strength of knee flexion-extension, flexion-abduction-external rotation (FAE), extension-adduction-internal rotation (EAI) upper extremity patterns in both extremities. All test protocols provided by the manufacturers were strictly followed. The angular velocity was adjusted to 60°/sec and 120°/sec, as recommended in previous studies and as can be tolerated by AIS patients [29, 30]. To assess the flexion-extension muscle strength of both knees, the patients were placed in a sitting position with arms lying against the body, hands holding lateral fixed handles, and the tested leg stabilized by a belt on the thigh. During testing of the strength of the upper extremity patterns, patients were placed in a sitting position with one arm lying against the body, one hand holding a lateral fixed handle, and the trunk stabilized by belts. Three repetitive adaptation periods were applied at an angular velocity of 60°/sec in order to increase the adaptation of the participants to the device before measurements were taken at both 60°/sec and 120°/sec angular velocity. Five minutes after the adaptation period, muscle strength tests were performed using five repetitions at an angular velocity of 60°/sec and 10 repetitions at an angular velocity of 120°/sec. Rest periods of five minutes were given between the tests of each muscle group and the velocity trials to prevent fatigue [30]. The patients were asked to perform the exercise as forcefully and fast as possible, and they were verbally encouraged during the process. Peak torque/body weight (Nm/kg) was recorded as an outcome parameter. Studies have shown that isokinetic muscle testing is useful for assessing scoliosis and should be included in the determination of treatment strategies [29, 30].

During the initial period of our study, the gait parameters of most of the participants were evaluated. Unfortunately, the device broke down before all participants could be assessed. Despite all efforts, the device could not be repaired within the required timeframe. Thus, this parameter was omitted because no similar device was found in the province where the study was conducted. We subsequently informed the ethics committee about this issue.

### Sample size

To determine the sample of the study, version 3.1.9.4 of the G*Power program (Heinrich-Heine-Universität Düsseldorf, Germany) was used [31]. According to previous studies, the effects of exercises on the Cobb angle of the main curve were determined to be from small to moderate (0.16–0.38) [32, 33]. To obtain 80% statistical power (1 − β error probability) with an α error level probability of 0.05, we performed repeated measure analysis of variance (ANOVA) within and between interactions, used a medium effect size of 0.30 to consider the two groups, and used two measurements for the primary outcome, generating a sample size of 24 participants. Considering the drop-out rate of 15% and aiming to increase the statistical power of the results, a total of 28 participants (14 for each group) were recruited into the study.

### Randomization and blinding

A randomization process was performed for the 28 AIS patients. The participants were randomly divided into two groups as SG and CG using matched pairs randomization based on their Cobb angle, age, and gender. Matched pairs randomization was performed with numbers sorted using the Research Randomizer program on the *randomizer*.*org* website [34]. At baseline and after the 10-week treatment period, all assessments were evaluated by the investigator, who was blinded to the groups throughout the study (M.H.K.).

### Statistical analysis

Statistical analysis was performed using IBM^®^ SPSS (Version 21.0 software, IBM Corp., Armonk, NY, USA). According to analytical methods (Kolmogorov–Smirnov and Shapiro–Wilk tests), the data were normally distributed. Thus, parametric tests were used for the statistical analysis. Descriptive statistics were presented as mean ± standard deviation (SD) or number and frequency. For comparison of demographic and subject characteristics between the two groups, the chi-square test was used for categorical variables, and the t-test was used for continuous variables. To analyze the changes within the groups over time and group–time interactions for continuous variables, a two-way mixed design repeated measures analysis of variance was performed. Based on Box’s M test, the assumption of the equality of covariance matrices, which is an important assumption in this test, was not violated. Also, pairwise comparisons, known as Bonferroni corrections, were performed for subsequent multiple comparisons. Partial eta squared was considered as the effect size. The effect size values considered were 0.10 = small, 0.25 = medium, and 0.40 = large [35]. Statistical significance was determined at a p value of < 0.05.

## Results

**Fig 1:**
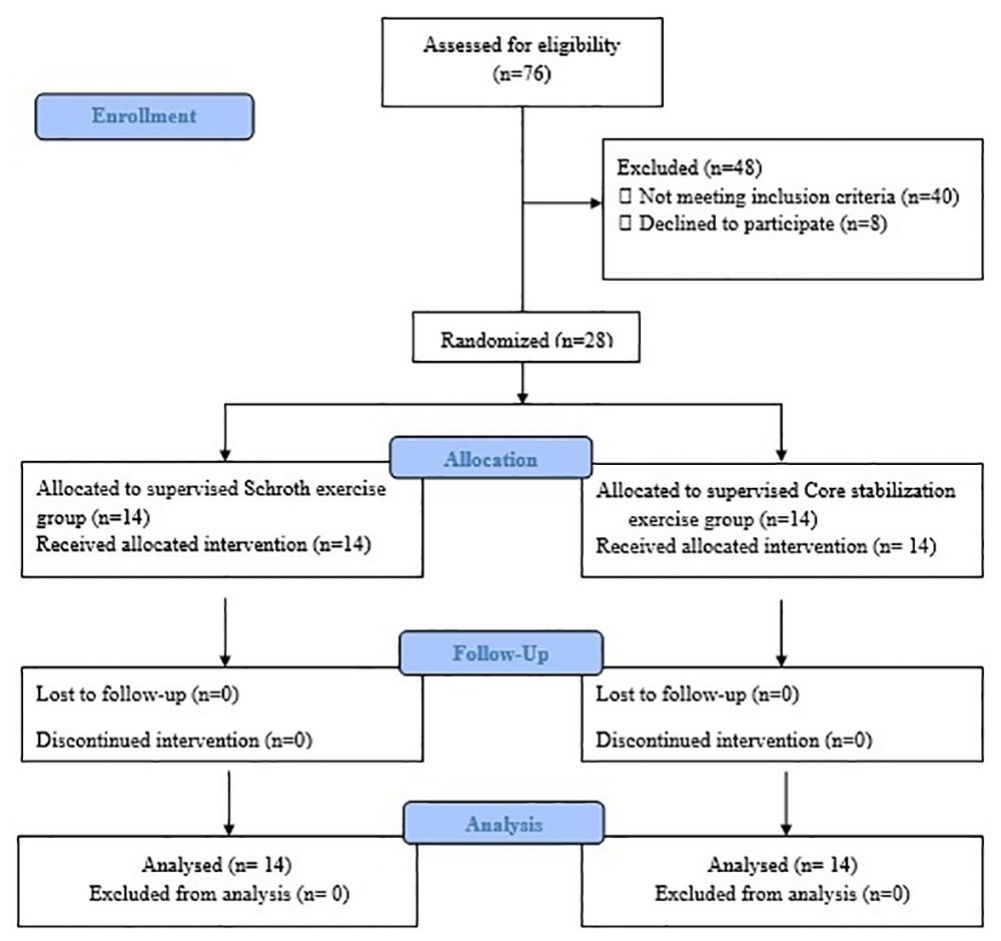
Study flow chart.

Seventy-six scoliosis patients were admitted to the department, and 28 (21 females, 7 males) satisfied the inclusion criteria. The patient distributions were n = 14 for the SG and n = 14 for the CG after randomization. The study was completed with 100% attendance compliance. The flow chart of the study is shown in Fig 1.

Baseline clinical and demographic characteristics of the patients are shown in Table 1. There was no significant difference between the two groups in terms of age, gender, body mass index, Risser sign, curve type, or dominant sides (p>0.05).

**Table 1:** Baseline clinical and demographic characteristics of groups.

		SG	CG	*p*
		(n = 14)	(n = 14)	
		Mean±SD	Mean±SD	
**Age (years)**		14.07**±**2.37	14.21±2.19	0.87
**BMI (kg/m**^**2**^**)**		19.77±4.12	19.73±2.36	0.98
**Risser sign**		1.64±1.34	1.78±1.19	0.77
		**n**	**n**	
**Gender**	Female	10 (71.4%)	11 (78.6%)	0.66
	Male	4 (28.6%)	3 (21.4%)	
**Dominant side (upper extremity)**	Right	14 (100.0%)	12 (85.7%)	0.14
	Left	0 (0.0%)	2 (14.3%)	
**Dominant side** (**lower extremity**)	Right	13 (92.9%)	13 (92.9%)	1.00
	Left	1 (7.1%)	1 (7.1%)	
**Curve type**	Right Thoracic	3 (21.4%)	3 (21.4%)	1.00
	Left Thoracic	5 (35.7%)	5 (35.7%)	
	Right Thoracic-Left lumbar	6 (42.9%)	6 (42.9%)	

SG: Schroth group, CG: Core group, SD: Standard deviation, BMI: Body-mass index.

There were significant group-by-time interactions for Cobb angles (thoracic: F = 32.39, p <0.001, η^2^ = 0.55 and lumbar: F = 19.47, p = 0.002, η^2^ = 0.68), WRVAS (F = 20.47, p<0.001, η^2^ = 0.44), thoracic angle of trunk rotation (ATR-T) (F = 35.61, p<0.001, η^2^ = 0.57), SRS-22 (F = 17.44, p<0.001, η^2^ = 0.40), total FP (F = 17.19, p<0.001, η^2^ = 0.39), and SP motion (F = 27.26, p<0.001, η^2^ = 0.51). There were no significant group-by-time interactions between the groups for the lumbar angle of trunk rotation (ATR-L) (F = 1.10, p = 0.302, η^2^ = 0.04). Within and between-group differences for the baseline and final treatment measurements for these parameters are shown in Table 2. Patients in the SG demonstrated a greater improvement in these parameters than those in the CG, except for in ATR-L.

**Table 2:** Baseline, post-ıntervention and change scores for the Cobb angle, WRVAS, ATR, SRS-22, total FP and SP motion.

	Baseline		Post treatment		Within-group change scores		Between-group difference in change scores	Group×time	
	SG	CG	SG	CG	SG	CG			
	Mean ± SD	Mean ± SD	Mean ± SD	Mean ± SD	Mean	Mean	Mean	F/ *p value*	η^2^
					(SE)	(SE)	(95% CI)		
Cobb-T (°)	17.64±4.01	17.29±3.45	9.71±3.47	13.57±5.03	-7.93	-3.71	4.21	32.39/ <0.001	0.55
					(0.47)	(0.57)	(2.69, 5.74)		
Cobb-L (°)	15.80±3.42	15.17±4.02	9.40±2.61	12.33±4.37	-6.40	-2.83	3.57	19.47/ 0.002	0.68
					(0.68)	(0.48)	(1.74, 5.39)		
WRVAS (7–35 points)	15.86±2.48	15.43±1.79	8.71±1.64	11.14±2.44	-7.14	-4.29	2.86	20.47/ <0.001	0.44
					(0.50)	(0.38)	(1.56, 4.16)		
ATR-T (°)	8.71±2.37	8.43±2.50	3.64±1.91	5.79±3.02	-5.07	-2.64	2.43	35.61/ <0.001	0.57
					(0.34)	(0.23)	(1.59, 3.27)		
ATR-L (°)	4.29±2.73	4.43±2.38	1.93±1.21	2.64±2.21	-2.36	-1.79	0.57	1.10/ 0.302	0.04
					(0.52)	(0.16)	(-0.58, 1.73)		
SRS-22 (0–5 points)	3.49±0.13	3.48±0.24	4.56±0.13	4.30±0.17	1.07	0.82	-0.25	17.44/ <0.001	0.40
					(0.03)	(0.05)	(-0.37, -0.13)		
Total FP Motion (°)	69.43±14.16	68.64±12.22	84.29±15.46	77.43±13.86	14.86	8.79	-6.07	17.19/ <0.001	0.39
					(0.88)	(1.17)	(-9.08, -3.06)		
Total SP Motion (°)	114.50±11.95	116.29±20.29	141.36±9.02	130.71±21.63	26.86	14.43	-12.43	27.26/ <0.001	0.51
					(2.09)	(1.13)	(-17.32, -7.54)		

SG: Schroth group, CG: Core group, SD: Standard deviation, SE: Standard error, CI: Confidence interval, η^2^: Effect size, Cobb-T: Cobb angle of thoracic, Cobb-L: Cobb angle of lumbar, ATR-T: Thoracic angle of trunk rotation, ATR-L: Lumbar angle of trunk rotation, FP: Frontal plane, SP: Sagittal plane.

There were significant group-by-time interactions for the FAE upper extremity pattern in both extremities at angular velocities of 60°/sec and 120°/sec (left 60°/sec: F = 9.47, p = 0.005, η^2^: 0.26 and 120°/sec: F = 4.76, p = 0.038, η^2^: 0.15; right 60°/sec: F = 11.71, p = 0.002, η^2^: 0.31 and 120°/sec: F = 14.24, p = 0.001, η^2^: 0.35). There were no significant group-by-time interactions for the EAI upper extremity pattern in both extremities at both angular velocities (left 60°/sec: F = 2.15, p = 0.154, η^2^: 0.07 and 120°/sec: F = 3.55, p = 0.070, η^2^: 0.12; right 60°/sec: F = 4.25, p = 0.051, η^2^:0.14 and 120°/sec: F = 3.51, p = 0.072, η^2^:0.11). As indicated by mean difference change scores, patients who received CS exercises showed significantly greater improvements in FAE upper extremity pattern in both extremities compared to those who received the Schroth exercises (Table 3).

**Table 3:** Baseline, post-ıntervention and change scores for the EAI and FAE upper extremity patterns’ muscle strengths.

	Baseline		After treatment		Within-group change scores		Between-group difference in change scores	Group×time	
	SG	CG	SG	CG	SG	CG			
	Mean ± SD	Mean ± SD	Mean ± SD	Mean ± SD	Mean	Mean	Mean	F/ *p value*	η^2^
					(SE)	(SE)	(95% CI)		
Left EAI-UEP PT/BW 60°/sec	41.49±11.38	41.98±10.55	57.91±16.05	63.18±11.29	16.42	21.20	4.79	2.15/ 0.154	0.07
					(2.81)	(1.66)	(-1.92, 11.49)		
Left FAE- UEP PT/BW 60°/sec	43.39±7.56	39.67±10.20	58.86±13.69	67.06±12.81	15.48	27.39	11.92	9.47/ 0.005	0.26
					(3.22)	(2.15)	(3.96, 19.87)		
Left EAI- UEP PT/BW 120°/sec	45.73±14.79	42.51±11.23	60.80±15.84	63.25±10.96	15.08	20.73	3.47	3.55/ 0.070	0.12
					(2.63)	(1.44)	(-5.46, 12.39)		
Left FAE- UEP PT/BW 120°/sec	41.89±7.98	40.86±8.73	60.82±11.73	68.00±13.85	18.93	27.14	8.20	4.76/ 0.038	0.15
					(3.01)	(2.25)	(0.47, 15.93)		
Right EAI- UEP PT/BW 60°/sec	47.13±8.77	44.16±13.38	64.26±12.67	67.01±12.25	17.13	22.86	5.73	4.25/ 0.051	0.14
					(2.22)	(1.67)	(0.02, 11.44)		
Right FAE- UEP PT/BW 60°/sec	46.93±10.74	39.92±7.89	64.63±16.21	70.71±14.86	17.70	30.79	13.08	11.71/ 0.002	0.31
					(3.03)	(2.33)	(5.23, 20.94)		
Right EAI- UEP PT/BW 120°/sec	47.11±13.34	44.23±12.99	64.02±15.81	67.17±12.81	16.91	22.95	6.04	3.51/ 0.072	0.11
					(2.76)	(1.66)	(-0.59, 12.66)		
Right FAE- UEP PT/BW 120°/sec	44.52±10.84	41.82±10.19	61.41±12.46	70.06±13.86	16.89	28.24	11.35	14.24/ 0.001	0.35
					(2.28)	(1.96)	(5.17, 17.53)		

SG: Schroth group, CG: Core group, SD: Standard deviation, SE: Standard error, CI: Confidence interval, η^2^: Effect size, EAI: Extension-adduction-internal rotation, FAE: Flexion-abduction-external rotation, UEP: Upper extremity pattern, PT/BW: Peak torque/body weight.

There were significant group-by-time interactions for the muscle strength of the left knee extensor (F = 28.76, p = <0.001, η^2^:0.52) and the right knee flexor (F = 15.92, p<0.001, η^2^: 0.38) at an angular velocity of 60°/sec. There were significant group-by-time interactions for the muscle strengths of the knee flexor (left: F = 21.15, p = <0.001, η^2^:0.44; right: F = 9.84, p<0.001, η^2^:0.27) and extensor (left: F = 26.79, p<0.001, η^2^:0.50; right: F = 39.46, p<0.001, η^2^: 0.60) in both extremities at an angular velocity of 120°/sec. There were no significant group-by-time interactions for the muscle strength of the left knee flexor (F = 2.89, p = 0.101, η^2^:0.10) and right knee extensor (F = 1.00, p = 0.326, η^2^:0.03) at an angular velocity of 60°/sec. As indicated by mean difference change scores, patients treated with CS exercises showed significantly greater improvements in knee flexor-extensor muscle strength in both extremities than those who were treated with the Schroth exercises, except in muscle strength of the left knee flexor (angular velocity of 60°/sec) and right knee extensor (angular velocity of 60°/sec) (Table 4).

**Table 4:** Baseline, post-intervention and change scores for the knee flexor-extensor muscle strengths.

	Baseline		After treatment		Within-group change scores		Between-group difference in change scores	Group×time	
	SG	CG	SG	CG	SG	CG			
	Mean ± SD	Mean ± SD	Mean ± SD	Mean ± SD	Mean	Mean	Mean	F/ *p*	η^2^
					(SE)	(SE)	(95% CI)		
Left KF PT/BW 60°/sec	102.20±17.35	100.03±22.22	121.34±18.71	125.09±21.15	19.13	25.06	5.92	2.89/ 0.101	0.10
					(3.06)	(1.65)	(-1.23, 13.08)		
Left KE PT/BW 60°/sec	167.55±28.66	158.05±36.04	185.95±29.97	199.69±32.66	18.40	41.65	23.25	28.76/ <0.001	0.52
					(2.04)	(3.83)	(14.34, 32.29)		
Left KF PT/BW 120°/sec	79.79±19.56	77.27±19.64	93.94±19.41	101.92±18.83	14.16	24.65	10.49	21.15/ <0.001	0.44
					(1.42)	(1.79)	(5.80, 15.18)		
Left KE PT/BW 120°/sec	108.61±28.76	112.57±27.66	129.49±26.27	152.81±25.58	20.88	40.24	19.36	26.79/ <0.001	0.50
					(2.49)	(2.79)	(11.67, 27.05)		
Right KF PT/BW 60°/sec	104.10±24.11	104.43±17.90	120.40±22.13	131.40±20.61	16.29	26.98	10.68	15.92/ <0.001	0.38
					(1.40)	(2.28)	(5.18, 16.18)		
Right KE PT/BW 60°/sec	170.87±35.33	170.86±33.89	210.43±42.04	216.46±34.86	39.56	45.59	6.04	1.00/ 0.326	0.03
					(4.14)	(4.38)	(-6.35, 18.42)		
Right KF PT/BW 120°/sec	78.58±21.09	85.28±14.15	93.66±19.61	109.62±17.34	15.08	24.34	9.26	9.84/ <0.001	0.27
					(2.02)	(2.15)	(3.19, 15.32)		
Right KE PT/BW 120°/sec	115.23±27.22	121.04±20.53	135.68±22.14	164.81±20.82	20.46	43.77	23.31	39.46/ <0.001	0.60
					(2.68)	(2.56)	(15.68, 30.94)		

SG: Schroth group, CG: Core group, SD: Standard deviation, SD: Standard error, CI: Confidence interval, η^2^: Effect size, KF: Knee flexor, KE: Knee extensor, PT/BW: Peak torque/body weight.

In addition, pairwise comparisons revealed significant differences between pretreatment and posttreatment for Cobb-T (p<0.001), Cobb-L (p = 0.001 in SG; p = 0.002 in CG), WRVAS (p<0.001), ATR-T (p<0.001), ATR-L (p = 0.001 in SG; p<0.001 in CG), SRS-22 (p<0.001), total FP and SP motion (p<0.001), left and right EAI and FAE upper extremity patterns’ muscle strengths (p<0.001), and left and right knee flexor-extensor muscle strengths (p<0.001) in both intervention groups.

## Discussion

To the best of our knowledge, the present study is the first to compare these two exercise methods used in the conservative treatment of AIS. This single-blind, randomized controlled study indicated that the Schroth exercises are superior in improving Cobb angles, ATR-T, spinal mobility, cosmetic trunk deformity, and quality of life compared to CS exercises in patients with AIS after a 10-week treatment. Improvement in the ATR-L was similar in both groups, and CS exercises were superior in the improvement of peripheral muscle strength.

In the study of AIS, Cobb angle and ATR are major prognostic and clinical indications of curve progression [36]. Strategies that slow the progression of scoliosis and decrease the need for surgery are important in the treatment of AIS [37]. In the present study, the Cobb angle and ATR of the scoliotic curve decreased in all participants. However, patients in the SG had a greater decrease in Cobb angle and ATR than those in the CG, except in lumbar trunk rotation angle. These findings suggest that the Schroth exercise program is superior to the CS exercise program in reducing Cobb angle or ATR. Similar to the results presented here, a systematic review reported that corrective, therapeutic exercise based on the Schroth method or involving CS exercises can reduce vertebral angles and body asymmetries [17]. Kuru et al. [32] found that the Schroth exercise program applied in the clinic under the supervision of a physiotherapist decreased the Cobb angle and ATR, and Gür et al. [15] found that a 10-week CS exercise program decreased Cobb angles (thoracic and lumbar) and ATR in patients with AIS. It has been established that ATR is associated with curve magnitude and directly affects Cobb angle [38]. In the present study, the reduction in the ATR, as it relates to the decrease in Cobb angle, is consistent with the literature.

A main concern of scoliosis patients is anxiety developed due to three-dimensional deformity [39]. Correction of cosmetic deformity is a primary goal of treatment, as reported in a consensus by SOSORT [4]. Therefore, measuring the perception of cosmetic problems is important for patients, their families, and clinicians in order to evaluate the outcome of treatment [23]. WRVAS has been found to be sensitive to improvement or worsening of the deformity of scoliosis [23, 24]. A study on treatments using CS exercise or the scientific exercise approach to scoliosis (SEAS) found both approaches considerably improve cosmetic deformity in moderate AIS [40]. Another study reported that when the Schroth exercise program was added to standard care, the body image of patients with AIS improved [9]. A systematic review on this subject reported that corrective, therapeutic exercises, such as the Schroth method or CS exercises, improved body symmetry in patients with AIS [17]. In the present study, cosmetic deformity was evaluated using WRVAS, and the cosmetic deformities of both groups improved considerably. However, patients in the SG had greater cosmetic improvement than those in the CG. The greater cosmetic deformity improvement in the SG may be the result of greater improvement in body symmetry and greater reduction in curve magnitudes.

Spinal flexibility and mobility decrease in AIS patients due to structural deformity of the spine, which can become more rigid over time [41]. Kao et al. [42] found a negative correlation between Cobb angle, vertebral rotation, and results of the sit-and-reach test in individuals with AIS and determined that a greater Cobb angle and vertebral rotation lead to greater restriction of lumbar flexion. In conservative treatment, increasing the flexibility and mobility of the spine is important to correct the curve of the spine in scoliosis [43]. The effect of the Schroth method on the flexibility and mobility of the spine has been documented in previous studies [7, 44, 45]. Malaj et al. [44] reported an improvement in trunk flexion after treatment with combined Schroth and Pilates exercises in patients with AIS. Another study examined the effects of CS exercise on flexibility in scoliosis and showed that lumbar flexibility increased significantly after CS exercise in patients with AIS [13]. Consistent with the previous studies, FP and SP mobility increased in both groups in our study. However, the Schroth exercise program produced more improvement in FP and SP mobility compared to the CS exercise program. This result may be attributed to a greater increase in the flexibility of the curve with the Schroth exercise program.

Depending on the severity of the curve, physical and psychological problems can arise in patients with AIS [36, 39]. Different results have been reported in the literature regarding the effect of exercise on quality of life in scoliosis patients. Some studies have reported that different exercise protocols have positive effects on quality of life, while some studies have not found any effect from exercise on improving the quality of life of patients with AIS [32, 46–48]. The findings of the present study support the hypothesis that exercise promotes a positive quality of life. However, in our study, patients in the SG showed more improvement in quality of life than those in the CG.

Generalized muscle dysfunction is common in patients with AIS and contributes to limitations in their exercise capacity, even in the absence of severe ventilatory impairment [39, 49, 50]. Several studies have reported on the effect of Schroth or CS exercise on the strength of trunk muscles in patients with AIS [10, 13, 45, 51]. Screiber et al. [9] demonstrated that adding the Schroth exercise program to standard care improves back muscle endurance in AIS patients, and Otman et al. [10] found that trunk muscle strength increased significantly after a six-week Schroth exercise program. Another study reported that lumbar muscle strength improved after 12 weeks of CS exercise in AIS patients [13], and a recent meta-analysis determined that the Schroth exercise program mostly influences core muscle strength [7]. In the present study, peripheral muscle strength increased in both groups. However, the peripheral muscle strength improvement of patients in the CG was greater than that of those in the SG. We could not find a study that examined the effect of exercise therapies on peripheral muscle strength in AIS with which we could compare our results. To the best of our knowledge, the present study is the first to compare the effect of two different exercise methods on peripheral muscle strength in patients with AIS.

This study has several limitations. First, only the patients who had Lenke curve type 1 were recruited for the study. Second, the findings of this study are only for a 10-week treatment program. Third, although the inclusion criterion for Cobb angle was 10–30°, most of the patients included in the study had mild curve magnitude. Therefore, this study could not be generalized to other types of scoliotic curves, different curve magnitudes, and different treatment periods. Future studies should take these into consideration.

## Conclusion

The present study indicated that Schroth exercises are more effective in reducing Cobb angle and ATR (main curve) and in improving spinal mobility and quality of life in patients with mild AIS than are CS exercises, while CS exercises are more effective than Schroth exercises in the improvement of peripheral muscle strength. Both exercise methods can be used in the conservative treatment of mild AIS, depending on the treatment’s purpose. Further studies are needed with long-term follow-up periods and patients with different curve types, curve magnitudes, and other scoliosis types (neuromuscular, early-onset, adult) and conditions.

## Supporting information

S1 Appendix Program of core stabilization exercises. (DOCX)

S2 Appendix Program of Schroth exercises. (DOCX)

S1 File Study protocol. (DOCX)

S2 File Study protocol-Turkish. (DOC)

S1 Checklist CONSORT 2010 checklist of information to include when reporting a randomised trial. (DOC)
