# Supplementary material for: The effectiveness of two different exercise approaches in adolescent idiopathic scoliosis: A single-blind, randomized-controlled trial
Source: PLoS One. 2021 Apr 15;16(4):e0249492. doi: 10.1371/journal.pone.0249492 (PMC8049223; doi:10.1371/journal.pone.0249492)
Supplement: S1 Appendix — (DOCX) [file pone.0249492.s001.docx]

**S1 Appendix.** Program of core stabilization exercises

| **Core stabilization exercises** | **Early Phase** | **Mid-Phase** | **Advanced Phase** |
| --- | --- | --- | --- |
|  | Learning activation of TrA and ML muscles in supine hook position | Warm up stretching | Warm up stretching |
|  | Training of the continuation of the neutral lumbopelvic control during exercises | Supine leg lift with yellow Theraband | Supine leg lift with red Theraband |
|  | Warm up stretching | Supine contralateral limb lift | Abdominal curl |
|  | Supine single leg lift | Supine bicycles | Supine bridge ball rolls |
|  | Supine flexed knee pull | Supine bridge single leg | Supine bridge; knee flexed and legs on the ball |
|  | Supine single arm lift | Supine bridge; knee extended and legs on the swiss ball | Prone bridging |
|  | Supine bridge | Supine ball rolls with legs | Side bridge |
|  | Clamshell | Side bridge with bent knee | Side leg lift with red Theraband |
|  | Side-lying leg lift | Side leg lift with yellow Theraband | Clamshell with red Theraband |
|  | Cat-camel | Clamshell with yellow Theraband | Cat-camel |
|  | Superman (arm) | Cross limb superman | Cross limb superman |
|  | Superman (leg) | Cat-camel | Seated cross limb raise on the swiss ball |
|  | Sitting on a swiss ball and pelvic tilt with core stabilization | Seated leg raise on the swiss ball | Seated arm raise on the swiss ball with red Theraband (PNF exercises) |
|  | Seated arm raise on the swiss ball (PNF exercises) | Seated arm raise on the swiss ball with yellow Theraband (PNF exercises) | Standing arm raise (PNF exercises) with red Theraband |
|  | Standing arm raise (PNF exercises) | Standing arm raise (PNF exercises) with yellow Theraband | Cool down stretching |
|  | Cool down stretching | Cool down stretching |  |
