# Supplementary material for: The effectiveness of two different exercise approaches in adolescent idiopathic scoliosis: A single-blind, randomized-controlled trial
Source: PLoS One. 2021 Apr 15;16(4):e0249492. doi: 10.1371/journal.pone.0249492 (PMC8049223; doi:10.1371/journal.pone.0249492)
Supplement: S2 Appendix — (DOCX) [file pone.0249492.s002.docx]

**S2 Appendix.** Program of Schroth exercises.

| **Schroth exercises** | **Early Phase** | **Mid-Phase** | **Advanced Phase** |
| --- | --- | --- | --- |
|  | 3D corrective breathing | 3D corrective breathing | 3D corrective breathing |
|  | Shoulder counter-traction in supine position | Shoulder counter-traction in sitting position | Shoulder counter-traction in sitting position |
|  | Shoulder counter-traction in prone position | Chest twister | Chest twister |
|  | Shoulder counter-traction in side-lying position | Muscle cylinder in sitting position | Muscle cylinder in kneeling position |
|  | Muscle cylinder in supine position | Big bow | Big bow |
|  | Muscle cylinder in side-lying position | Shoulder counter-traction between two poles | Shoulder counter-traction between two poles |
|  | Muscle cylinder in sitting position | Schroth gait | Schroth gait |
|  | Chest twister | Removing the stool | Removing the stool |
