# Supplementary material for: The effectiveness of two different exercise approaches in adolescent idiopathic scoliosis: A single-blind, randomized-controlled trial
Source: PLoS One. 2021 Apr 15;16(4):e0249492. doi: 10.1371/journal.pone.0249492 (PMC8049223; doi:10.1371/journal.pone.0249492)
Supplement: S1 File — (DOCX) [file pone.0249492.s003.docx]

**Document Date:** 18.09.2019

**Version No:** 2

**Name of the Research Project:** "Investigation of the Effects of Two Different Exercise Methods on Scoliosis Severity, Gait, Physical, Functional Parameters and Quality of Life in Adolescent Idiopathic Scoliosis"

**Coordinator:** Mehmet YETİŞ

**Researchers:** Hikmet KOCAMAN

Nilgün BEK

Mehmet Hanifi KAYA

Buket BÜYÜKTURAN

Öznur BÜYÜKTURAN

**Place of Implementation of the Project:** Kırşehir Ahi Evran University School of Physical Therapy and Rehabilitation

**Research Design:** Randomized Controlled Single-Blind Study

**Estimated Research Time:** 6 month

**Introduction and purpose**

Adolescent idiopathic scoliosis (AIS) is a progressive growth disease with unknown etiology, characterized by a three-dimensional deformity of the spine (frontal translation, sagittal modification, and torsion of the spinous processes on the concave side of the scoliotic curve on radiographs). In patients with AIS, in addition to curve progression there are many problems commonly occur such as; muscular imbalance, functional limitations, altered posture, gait deviations, reduced flexibility of the spine, back pain, negative physico-social, body image effects, and in severe cases pulmonary symptoms. The asymmetry of the trunk and pelvis are affected related to the shape and angle of scoliosis, and the weight distribution position changes depending on the shape and the Cobb angle of scoliosis. To deal with these complications and more, various treatment approaches have been proposed for AIS, including exercise, bracing, casting, traction, biofeedback, surgery, and simple observation to prevent, correct or halt the progression of the deformity. Conservative treatment methods including physiotherapy and bracing are accepted in Central Europe.

In literature, exercises are recommended to decrease progression, to improve spine and thoracic cage flexibility, muscle strengths, and elasticity, to correct postural behavior, and neuro-motor control, spine stability. In general, traditional exercises (TE) including postural training, stretching, and strengthening exercises for spinal musculature, respiratory exercises have been used for many years for scoliosis. Except for the traditional exercises, there are several exercise concepts including Schroth, Side Shift, Dobomed methods... etc. Schroth exercises are asymmetric scoliosis-specific postural exercises that aim to improve the curve, function, posture, self-image, and pain. Schroth exercises target strength and endurance training of the back, abdominal, and leg muscles. Also one of the aim of Schroth exercises is to improve motor control of the posture by repeating corrective movements with progressively less feedback. Schroth exercises are the most studied scoliosis exercises but there are limited randomized controlled studies on Schroth exercises. Recently general physiotherapeutic exercises including, Core stabilization (CS) exercises, Pilates have been used in the conservative treatment of idiopathic scoliosis. CS exercises are described as therapy techniques that improve postural control, and functional stability through increasing neuromuscular control, the strength of trunk stabilization muscles, the endurance of postural muscles around the spine, the balance between pelvis and spine. However, limited studies are determining CS exercises' effect on patients with AIS. In a study CS exercises found to be more effective in reducing pain and rotational deformity than traditional exercises in the conservative rehabilitation of AIS. A recent systematic review showed that therapeutic exercise had been effective to reduce symptoms, Cobb’s angle, trunk rotation, craniovertebral angle, and body asymmetries, and to improve muscular endurance, pulmonary function, and functional capacity of patients with AIS. Corrective, therapeutic exercises appear to have positive effects by improving function and reducing symptoms, as well as various angles and body asymmetries. However, further studies with better methodological quality are required to confirm these outcomes and detect the best therapeutic exercise intervention. And also there is a need for randomized controlled studies on different methods of exercise to choose the most effective exercise in clinical practice.

There was no research compared to the effects of the Schroth method and CS exercises in patients with AIS. And also there was no research examining the effects of the Schroth method on peripheral muscle strengths. This study aimed to investigate the effects of the Schroth versus CS exercises in addition to traditional exercises, on Cobb angle, trunk rotation, peripheral muscle strengths, spine mobility, cosmetic deformity, gait, and health-related quality of life in patients with AIS.

**Materials and Methods**

In this study, the effects of two different exercise methods on the severity of scoliosis, pedobarographic gait parameters, physical and functional parameters and quality of life in Adolescent Idiopathic Scoliosis (AIS) will be examined. According to the results of the power analysis, 28 individuals with AIS will be included in the study and divided into two groups by paired randomization. Core stabilization exercises will be applied to one group in addition to conventional exercises, while Schroth exercises, one of the three-dimensional exercises specific to scoliosis, will be applied to the other group in addition to conventional exercises. Within the scope of the study, the demographic information of the adolescents with type 1 curvature according to the Lenke classification will be taken, and the bone maturation levels according to Risser and the spine regions containing the curve will be recorded.

Cobb angles of curves with anteroposterior X-ray; trunk rotations with scoliometer in forward bend test; with the Biodex System 4 Pro® isokinetic device, upper extremity pattern and lower extremity isokinetic muscle strengths; spinal mobility with a spinal mouse; cosmetic deformity perceptions according to Walter Reed Visuel Assessment Scale (WRVAS); quality of life according to the SRS-22 questionnaire; pedobarographic gait parameters will be evaluated with DIASU Digital Analysis System® and Milletrix software (DIASU, Italy) before and after the treatment program including 10-week Schroth and core stabilization exercise program.

Individuals who applied to Ahi Evran University Training and Research Hospital Orthopedics and Traumatology Outpatient Clinic will be included in the study. If individuals meet the inclusion criteria and volunteer to participate in the study, they will be directed to Ahi Evran University School of Physical Therapy and Rehabilitation. 28 individuals will be randomly divided into 2 groups with the paired randomization system in terms of age, curvature degree and Risser's finding. Before starting our study, approval will be obtained from the Ahi Evran University clinical research ethics committee and the study will be conducted in accordance with the Helsinki declaration.

All patients participating in the study and their families will be informed verbally and in writing about the content of the study and the way it is applied, participant rights, and the questionnaires to be used, and their consent will be obtained.

**Inclusion nd Exclusion Criteria**

*Inclusion Criteria;*

-Ages between 10-18 and diagnosed with AIS

-Not using Brace,

-Cobb angle between 10º-30º,

-Have a Lenke type 1 curvature

-Ability to continue the program to be applied,

-No pulmonary or chest-related diseases such as costa fracture, atelectasis, asthma.

-No neurological or psychiatric illnesses and no chronic illnesses requiring medication,

-Parents allow the child to participate in the study.

*Exclusion Criteria;*

-Non-idiopathic scoliosis

-Prescribed brace

-Surgical correction history

-Who were unable to participate in the supervised sessions, or those who refused to follow treatment were excluded.

-Contraindications to exercise -accompanying mental problems, neurological- muscular or rheumatic diseases,

**Data Collection Tools and Features**

**1)** Sociodemographic evaluation

- Age, height, weight, body mass index, background, family history, dominant side, Risser's sign

**2)** Cobb angle of the degree of curvature

**3)** Measuring spinal mobility with a spinal mouse

**4)** Measuring by scoliometer to determine the degree of trunk rotation

**5)** Upper extremity pattern (flexion-abduction-external rotation / extension-adduction-internal rotation) muscle strength and isokinetic force measurements in the determination of quadriceps and hamstring muscle strengths in the lower extremities.

**6)** Evaluating the perception of cosmetic deformities with Walter Reed Visual Assessment Scale (WRVAS)

**7)** Evaluation of quality of life with Scoliosis Research Society-22 questionnaire

**8)** Evaluation of gait parameters will be carried out with DIASU Digital Analysis System® and Milletrix software (DIASU, Italy).

**Socio-Demographic Evaluation**

Individuals' age, body weight and height, anamnesis, medical history, age of menarche, dominant hands and feet, exercise habits (previous sports) and their duration will be questioned and recorded.

**Risser's sign (Illiac apophysis):** It was first described by Joseph C. Risser in 1958. Risser observed that the ossification state of the iliac apophysis is related to the developmental state of the spinal skeleton and stated that it is a critical information in the treatment of AIS. According to the ossification of the iliac apophysis from anterolateral to posteromedial, it is evaluated by grading between 0 and 5 (0: no bone fusion started; 5: bone fusion completed). Risser's sign is used to determine the age of bone growth, growth rate and the risk for scoliosis. Risser grade is directly related to the progression of the curvature, and the risk of scoliosis progression decreases as skeletal maturation is complete. It will be used in our study to determine the degree of bone maturation.

**Cobb Angle:** It enables the evaluation of the coronal plane deformity in scoliosis. The lateral flexion angle of scoliosis is measured from the antero-posterior standing spine x-ray containing the whole spine. The Cobb method is accepted as the gold standard method of measurement in determining the degree of curvature. The Cobb angle describes only one plane of a three-dimensional deformity, but gives information about the progression of the curvature. Cobb angle will be measured on the basis of the same vertebral endplates on radiographs taken twice in total, at the beginning and after 10 weeks of treatment. In our study, the degree of change according to the Cobb angle will be examined to evaluate the effect of 10-week treatments.

**Lenke Classification:** This classification has three components: six curvature types, lumbar spine markers, and thoracic spine markers. First of all, the location of the curvature is determined as proximal thoracic, thoracic, thoracolumbar or lumbar. Then the deformity with the largest Cobb angle is called major curvature, and others are called minor curvature. Curvatures without elasticity are called structural curvatures. In Lenke Type I, there is a major curvature in the main thoracic. Curves in the proximal thoracic and lumbothoracal are minor and non-structural. There are double thoracic curves in Lenke Type II curves. Curves in the proximal thoracic and main thoracic are structural, while the curvature in the lumbothoracal is not structural. In Lenke Type III, there is a basic curvature in the main thoracic and structural curvature in the thoracolumbar. Triple curvatures are Lenke Type IV. In Lenke Type V, the main curvature is in the thoracolumbar junction. Curves in the proximal thoracic and main thoracic are not structural. In Lenke Type VI, the curvature in the main thoracic and thoracolumbar / lumbar region is structural and is greater than the curvature in the main thoracic. Individuals with Lenke Type I curvature will be included in our study.

**Cosmetic deformity perception:** Walter Reed Visual Assessment Scale (WRVAS) will be used to evaluate individuals' perception of cosmetic deformities and to evaluate the effectiveness of treatment in improving body cosmetic deformity. The WRVAS is divided into 7 parameters, which show 5 different figures including body curvature, prominence of the rib, prominence of lumbar prominence, head-rib-pelvis positional relationship, head-pelvis relationship, shoulder level and scapular rotation. Each parameter is scored increasingly from 1 to 5. The person marks what is suitable for his body between 1-5. It scores the curve intensity by focusing on the individuals' posture perception.

**Assessment of health-related quality of life:** The Scoliosis Research Society-22 questionnaire (SRS-22) is a health-related quality of life questionnaire designed specifically for patients with spinal deformities. SRS-22 includes 5 main topics: function, pain, mental health, body image and satisfaction with treatment. The score for each item ranges from zero (worst) to five (best). It will be used to evaluate the quality of life before and after treatment.

**Trunk Rotation Degree:** Measuring the rotation asymmetry of the trunk with a special inclinometer called scoliometer for the changes in the horizontal plane that develops due to scoliosis is a frequently used clinical method. While the interreliability of the scoliometer was found to be "excellent", it was stated that the reliability between measurements was "very good". The trunk rotation will be recorded in degrees by the physiotherapist by measuring the angle of rotation of the apex vertebra in the forward bending test with scoliometer.

**Muscle Strength:** Individuals on both sides upper extremity (flexion-abduction-external rotation-extension-adduction-internal rotation) pattern muscle strength and quadriceps, hamstring muscle strength will be evaluated with Biodex System 4 Pro® (Biodex Corp, Shirley, NY) device before, and after 10 weeks of treatments. Maximum isokinetic muscle strength will be measured as 5 repetitions at 60˚ / sec and 10 repetitions of 120˚ / sec.

**Spinal Mobility Assessment:** Evaluation of spinal mobility will be performed with a portable, computer-assisted electromechanical device (the Spinal Mouse System, Idiag, Fehraltorf, Switzerland) called Spinal Mouse (SM). The SM is an external, non-invasive measuring device that can evaluate spinal angles and curvatures in the frontal and sagittal planes. It has been reported that SM can be used as a reliable, fast and easy-to-use measurement method without side effects for clinical research and patient follow-up in AIS. Measurements will be made between the spinous process of the cervical 7th vertebra and the top of the anal crease (approximately the sacral 3rd vertebra level). Maximum right-left lateral flexion degrees in the frontal plane and maximum degrees of flexion-extension in the sagittal plane will be measured and recorded.

**Pedobarographic Gait Assessment:** Pedobarographic Gait Assessment: With the electronic sensors placed on a special platform and the computer system that analyzes the information from the sensors, the measurements of the condition of the foot and the loads on the foot can be performed at certain stages of walking. In our study, the static and dynamic pedobarographic gait analysis of individuals will be carried out with the DIASU Digital Analysis System® with 3x1 meter pressure sensor walking platform and Milletrix software (DIASU, Italy). In the static measurement, individuals are asked to stand in the position they feel comfortable and the percentage of contact in static conditions (%), maximal pressure (kg / cm2) and right-left foot separately; contact area (cm^2^), contact surface (%) values ​​will be recorded.

In dynamic measurements, individuals will be asked to walk three times at normal walking speed on the walking surface with pressure gauge sensors embedded in 3 m. By taking the averages of the data obtained from individuals, the loads (kg), average pressures (kg / cm^2^) affecting the fore-hind legs in dynamic conditions, separately for the right-left foot; contact area (cm^2^), contact surface (%) will be recorded.

**Traditional Exercises to be Applied in the Study**

The strengthening of the back and shoulder girdle muscles, stretching exercises for the concave side of the curve, postural training, flexibility exercises for the spine, and breathing exercises will be performed.

**Schroth Exercises to be Applied in the Study**

Among the individuals who accepted to participate in our study, those included in the Schroth group will be applied a 10-week Schroth exercise program, each session lasting approximately 90 minutes, together with traditional exercises, 3 days a week by an experienced physiotherapist who has received international Schroth 3D Scoliosis Therapy (ISST) training. In the Schroth method, exercises will be planned and applied individually to the structure of the curvature. First of all, the postural deviations caused by the primary curvatures and secondary curvatures, if any, and the breathing zones will be defined and the exercise program will be started by teaching the 3-dimensional corrective breathing exercise. Auxiliary materials such as a wall bar, mirror, exercise mat, 3 rice bags, sponge pillow, chair, and two long sticks will be used during the exercises. After the exercises are started in simple supine and side lying positions, they will be continued in increasingly difficult positions (sitting position, standing, walking). The exercises will be progressively progressed from 7-10 repetitions to 10-15 repetitions. Schroth exercises to be applied for 10 weeks are shown in Table 1.

**Table 1.** Schroth exercises to be applied in the study

| **Schroth exercises** | **Early Phase** | **Mid-Phase** | **Advanced Phase** |
| --- | --- | --- | --- |
|  | 3D corrective breathing | 3D corrective breathing | 3D corrective breathing |
|  | Shoulder counter-traction in supine position | Shoulder counter-traction in sitting position | Shoulder counter-traction in sitting position |
|  | Shoulder counter-traction in prone position | Chest twister | Chest twister |
|  | Shoulder counter-traction in side-lying position | Muscle cylinder in sitting position | Muscle cylinder in kneeling position |
|  | Muscle cylinder in supine position | Big bow | Big bow |
|  | Muscle cylinder in side-lying position | Shoulder counter-traction between two poles | Shoulder counter-traction between two poles |
|  | Muscle cylinder in sitting position | Schroth gait | Schroth gait |
|  | Chest twister | Removing the stool | Removing the stool |

**Corrective / Rotational Breathing:** In 3D deformity, by taking selective breathing into the concave areas collapsed under compression, the costal spaces are extended and the soft tissues are mobilized. The difference from spontaneous breathing is that the individual focuses on the compressed areas and inspiration locally on those areas. At first, hand contact and verbal notifications are used to direct the air taken to the right area. Later, the rotational breathing component is combined with all exercises, and postural correction is supported with rotational breathing exercises.

**Shoulder counter traction:** This exercise, in which deflection (bringing the lateral deviation of the spine closer to the midline) and derotation was tried to be achieved with the counter traction of the shoulder in order to correct the thoracic curvature, was applied in our study in the prone, supine, side lying and sitting position. While the thoracic convexity is pushed towards the midline during exhalation with rotational breathing, rotation is attempted against the rotation in the spine. At the same time, maximum correction is tried to be achieved with the counter traction of the shoulder on the convex side.

**Muscle Cylinder:** This exercise aims at deflection of the thoracic curvature, deflection of the lumbar curvature by pushing the heel caudal, and eccentric training of the quadratus lumborum muscle. It can be performed in side lying, half-kneeling position and standing. In side lying, the individual is placed on the thoracic concave side, the arm on the concave side is extended over the head to reduce the compression in the thoracic concavity, and a rice bag is placed in the lumbar region to correct the lumbar curvature. The shoulder on the convex side is retracted and the upper hand is placed on the pelvis and the lower extremity is extended caudally, while the foot is lifted slightly from the ground to try to correct the lumbar concavity.

**Removing the stool:** In this exercise, in which the back and surrounding muscles are tried to be strengthened with the elongation of the spine, the individual sits on a stool in front of the bar in a cross-legged position and holds the bar with arms. It makes basic corrections while breathing with a corrective/rotational breathing pattern and is asked to keep the position in 3-4 breathing time after removing the stool.

**Big bow:** In this exercise in which the spine is extended and elongated, the individual holds the bar in front of the bar and bends the knees slightly to reduce the tension of the hamstring muscles. In this position, thoracic, lumbar and pelvic corrections are made according to the type of curvature. While breathing out with a corrective / rotational breathing pattern, the arms are extended and thoracic flexion is performed. In this exercise, care should be taken to preserve kyphosis and lumbar lordosis during thoracic flexion.

**Between two poles:** Between two poles: This exercise aims to make basic corrections in the standing position with the counter-traction of the shoulder girdle using sticks. The sticks are positioned perpendicular to the ground, the convex side of the forearm adjacent to the stick, with the concave side arm straight, in the overhead position. After taking the exercise position, pelvic corrections (in the direction of shift, tilt, rotation) and correct weight transfer are started, axial elongation is performed and then with a corrective/rotational breathing, basic correction movements (deflection, derotation) are requested from the patient according to the type of curvature.

**Chest Twister:** In this exercise, it is tried to provide deflection and derotation in the thoracic region with the fixed pelvis. Basic corrections (sideshift, rotation) are applied as over-correction. While sitting on a chair facing the bar, the individual holds the bar by the arms in a diagonal position. It shifts its body to the thoracic concave side. Meanwhile, the lumbar concave side is positioned outside the pelvis chair for deflection of the lumbar concavity and correction of pelvic asymmetry. With corrective / rotational breathing, basic correction movements are repeated.

**Schroth gait**: In this exercise, in which the continuity and stabilization of corrections are aimed in dynamic conditions, corrections in three planes are made. After taking the exercise position and a corrective / rotational breathing, is asked to rise at the fingertip and perform maximum axial elongation while exhaling. It is repeated with every step forward.

**Core Stabilization Exercises to be Applied in the Study**

The Core Stabilization exercise program, which we have determined with reference to the core stabilization exercises applied in scoliosis in the literature, will be applied in 3 stages from easy to difficult. First, individuals were taught how to activate the trasversus abdominus muscle. In this direction, activation of the transversus abdominus muscle will be demonstrated by enabling them to palpate and feel the contraction of the transversus abdominus muscle with their hands placed on the anteromedial of the spina iliaca anterior superiors in the hook position in supine position. Later, patients will be asked to maintain the contraction of the transversus abdominus and multifidus muscles during exercises involving the activation of other muscles of the body in different positions. During all exercises, the importance of respiratory control will be emphasized, and they will be asked to perform movements by exhaling to prevent Valsalva maneuver, especially during the difficult component of the movement. Each movement will first be demonstrated by the physiotherapist and the patients will be corrected with verbal or tactile stimuli during the exercises so that they can perform the movements correctly. Each training session will consist of a 90-minute training program with traditional exercises that start with a 10-minute warm-up program and end with a 10-minute cooling program. The progression of the exercises will be personalized according to the capabilities of the patients. The exercises are designed to progress from 7-10 repetitions to 10-15 repetitions. In addition, exercises will be made gradually difficult by methods such as position changes, use of body weight, Theraband Elastic Band Hygienic Corporation, Akron, Ohio, and exercise ball.

The program, which is planned as three stages, is at initial level for the first 3 weeks, intermediate for the next 3 weeks, and advanced for the last 4 weeks. When patients complete the stage they started and move on to the next stage, the exercises will progress from less repetitions to more repetitions. When there is a movement that cannot be achieved in the next stage, the movement in the same program will be continued for a while and then the unreachable movement will be made. Core stabilization training to be applied to patients is shown in Table 2.

**Table 2.** Core stabilization exercises to be applied in the study

| **Core stabilization exercises** | **Early Phase** | **Mid-Phase** | **Advanced Phase** |
| --- | --- | --- | --- |
|  | Learning activation of TrA and ML muscles in supine hook position | Warm up stretching | Warm up stretching |
|  | Training of the continuation of the neutral lumbopelvic control during exercises | Supine leg lift with yellow Theraband | Supine leg lift with red Theraband |
|  | Warm up stretching | Supine contralateral limb lift | Abdominal curl |
|  | Supine single leg lift | Supine bicycles | Supine bridge ball rolls |
|  | Supine flexed knee pull | Supine bridge single leg | Supine bridge; knee flexed and legs on the ball |
|  | Supine single arm lift | Supine bridge; knee extended and legs on the swiss ball | Prone bridging |
|  | Supine bridge | Supine ball rolls with legs | Side bridge |
|  | Clamshell | Side bridge with bent knee | Side leg lift with red Theraband |
|  | Side-lying leg lift | Side leg lift with yellow Theraband | Clamshell with red Theraband |
|  | Cat-camel | Clamshell with yellow Theraband | Cat-camel |
|  | Superman (arm) | Cross limb superman | Cross limb superman |
|  | Superman (leg) | Cat-camel | Seated cross limb raise on the swiss ball |
|  | Sitting on a swiss ball and pelvic tilt with core stabilization | Seated leg raise on the swiss ball | Seated arm raise on the swiss ball with red Theraband (PNF exercises) |
|  | Seated arm raise on the swiss ball (PNF exercises) | Seated arm raise on the swiss ball with yellow Theraband (PNF exercises) | Standing arm raise (PNF exercises) with red Theraband |
|  | Standing arm raise (PNF exercises) | Standing arm raise (PNF exercises) with yellow Theraband | Cool down stretching |
|  | Cool down stretching | Cool down stretching |  |

**References**

1. Weinstein SL, Dolan LA, Cheng JCY, Danielsson A, Morcuende JA. Adolescent idiopathic scoliosis. The Lancet. 2008;371(9623):1527-37.

2. Tones M, Moss N, Polly Jr DW. A review of quality of life and psychosocial issues in scoliosis. Spine. 2006;31(26):3027-38.

3. Burgoyne W, Fairbank J. The management of scoliosis. Current Paediatrics. 2001;11(5):323-31.

4. Goldstein L, Waugh T. Classification and terminology of scoliosis. Clinical Orthopaedics and Related Research®. 1973;93:10-22.

5. James JIP. Idiopathic scoliosis: the prognosis, diagnosis, and operative indications related to curve patterns and the age at onset. The Journal of bone and joint surgery British volume. 1954;36(1):36-49.

6. Altaf F, Gibson A, Dannawi Z, Noordeen H. Adolescent idiopathic scoliosis. Bmj. 2013;346:f2508.

7. Lonstein J, Carlson J. The prediction of curve progression in untreated idiopathic scoliosis. J Bone Jt Surg. 1984:1061-71.

8. Sanders JO, Browne RH, Cooney TE, Finegold DN, McConnell SJ, Margraf SA. Correlates of the peak height velocity in girls with idiopathic scoliosis. Spine. 2006;31(20):2289-95.

9. Negrini S, Aulisa AG, Aulisa L, Circo AB, de Mauroy JC, Durmala J, et al. 2011 SOSORT guidelines: orthopaedic and rehabilitation treatment of idiopathic scoliosis during growth. Scoliosis. 2012;7(1):3.

10. Martínez-Llorens J, Ramirez M, Colomina M, Bagó J, Molina A, Cáceres E, et al. Muscle dysfunction and exercise limitation in adolescent idiopathic scoliosis. European Respiratory Journal. 2010;36(2):393-400.

11. Enneking W, HARRINGTON P. Pathological changes in scoliosis. JBJS. 1969;51(1):165-84.

12. Shea KG, Ford T, Bloebaum RD, D'astous J, King H. A comparison of the microarchitectural bone adaptations of the concave and convex thoracic spinal facets in idiopathic scoliosis. JBJS. 2004;86(5):1000-6.

13. Fidler M, Jowett R. Muscle imbalance in the aetiology of scoliosis. The Journal of bone and joint surgery British volume. 1976;58(2):200-1.

14. Zapata KA, Wang-Price SS, Sucato DJ, Dempsey-Robertson M. Ultrasonographic measurements of paraspinal muscle thickness in adolescent idiopathic scoliosis: a comparison and reliability study. Pediatric Physical Therapy. 2015;27(2):119-25.

15. Stetkarova I, Zamecnik J, Bocek V, Vasko P, Brabec K, Krbec M. Electrophysiological and histological changes of paraspinal muscles in adolescent idiopathic scoliosis. Eur Spine J. 2016;25(10):3146-53.

16. Martinez-Llorens J, Ramirez M, Colomina MJ, Bago J, Molina A, Caceres E, et al. Muscle dysfunction and exercise limitation in adolescent idiopathic scoliosis. The European respiratory journal. 2010;36(2):393-400.

17. Lin J-j, Chen W-H, Chen P-Q, Tsauo J-Y. Alteration in shoulder kinematics and associated muscle activity in people with idiopathic scoliosis. Spine. 2010;35(11):1151-7.

18. Bruyneel AV, Chavet P, Bollini G, Mesure S. Gait initiation reflects the adaptive biomechanical strategies of adolescents with idiopathic scoliosis. Annals of physical and rehabilitation medicine. 2010;53(6-7):372-86.

19. Bruyneel A-V, Chavet P, Bollini G, Allard P, Berton E, Mesure S. Dynamical asymmetries in idiopathic scoliosis during forward and lateral initiation step. Eur Spine J. 2009;18(2):188-95.

20. Daryabor A, Arazpour M, Sharifi G, Bani MA, Aboutorabi A, Golchin N. Gait and energy consumption in adolescent idiopathic scoliosis: A literature review. Annals of physical and rehabilitation medicine. 2017;60(2):107-16.

21. Lenssinck M-LB, Frijlink AC, Berger MY, Bierma-Zeinstra SM, Verkerk K, Verhagen AP. Effect of bracing and other conservative interventions in the treatment of idiopathic scoliosis in adolescents: a systematic review of clinical trials. Physical therapy. 2005;85(12):1329-39.

22. Romano M, Minozzi S, Zaina F, Saltikov JB, Chockalingam N, Kotwicki T, et al. Exercises for adolescent idiopathic scoliosis: a Cochrane systematic review. Spine. 2013;38(14):E883-E93.

23. Hawes MC. The use of exercises in the treatment of scoliosis: an evidence-based critical review of the literature. Pediatric rehabilitation. 2003;6(3-4):171-82.

24. Mordecai SC, Dabke HV. Efficacy of exercise therapy for the treatment of adolescent idiopathic scoliosis: a review of the literature. European Spine Journal. 2012;21(3):382-9.

25. Berdishevsky H, Lebel VA, Bettany-Saltikov J, Rigo M, Lebel A, Hennes A, et al. Physiotherapy scoliosis-specific exercises–a comprehensive review of seven major schools. Scoliosis and spinal disorders. 2016;11(1):20.

26. Bettany-Saltikov J, Parent E, Romano M, Villagrasa M, Negrini S. Physiotherapeutic scoliosis-specific exercises for adolescents with idiopathic scoliosis. Eur J Phys Rehabil Med. 2014;50(1):111-21.

27. Negrini S, Zaina F, Romano M, Negrini A, Parzini S. Specific exercises reduce brace prescription in adolescent idiopathic scoliosis: a prospective controlled cohort study with worst-case analysis. Journal of rehabilitation medicine. 2008;40(6):451-5.

28. Negrini S, Fusco C, Minozzi S, Atanasio S, Zaina F, Romano M. Exercises reduce the progression rate of adolescent idiopathic scoliosis: results of a comprehensive systematic review of the literature. Disability and rehabilitation. 2008;30(10):772-85.

29. Fusco C, Zaina F, Atanasio S, Romano M, Negrini A, Negrini S. Physical exercises in the treatment of adolescent idiopathic scoliosis: an updated systematic review. Physiotherapy theory and practice. 2011;27(1):80-114.

30. Kuru T, Yeldan I, Dereli EE, Ozdincler AR, Dikici F, Colak I. The efficacy of three-dimensional Schroth exercises in adolescent idiopathic scoliosis: a randomised controlled clinical trial. Clinical rehabilitation. 2016;30(2):181-90.

31. Schreiber S, Parent EC, Moez EK, Hedden DM, Hill D, Moreau MJ, et al. The effect of Schroth exercises added to the standard of care on the quality of life and muscle endurance in adolescents with idiopathic scoliosis-an assessor and statistician blinded randomized controlled trial: "SOSORT 2015 Award Winner". Scoliosis. 2015;10:24.

32. Ko K-J, Kang S-J. Effects of 12-week core stabilization exercise on the Cobb angle and lumbar muscle strength of adolescents with idiopathic scoliosis. Journal of exercise rehabilitation. 2017;13(2):244.

33. Akuthota V, Nadler SF. Core strengthening. Archives of physical medicine and rehabilitation. 2004;85:86-92.

34. Imai A, Kaneoka K, Okubo Y, Shiina I, Tatsumura M, Izumi S, et al. Trunk muscle activity during lumbar stabilization exercises on both a stable and unstable surface. Journal of orthopaedic & sports physical therapy. 2010;40(6):369-75.

35. Gür G, Ayhan C, Yakut Y. The effectiveness of core stabilization exercise in adolescent idiopathic scoliosis: A randomized controlled trial. Prosthetics and orthotics international. 2017;41(3):303-10.

36. Shin SS, Lee YW, Song CH. Effects of lumbar stabilization exercise on postural sway of patients with adolescent idiopathic scoliosis during quiet sitting. Journal of physical therapy science. 2012;24(2):211-5.

37. Reem J, Carney J, Stanley M, Cassidy J. Risser sign inter-rater and intra-rater agreement: is the Risser sign reliable? Skeletal radiology. 2009;38(4):371-5.

38. Horne JP, Flannery R, Usman S. Adolescent idiopathic scoliosis: diagnosis and management. Am Fam Physician. 2014;89(3):193-8.

39. Langensiepen S, Semler O, Sobottke R, Fricke O, Franklin J, Schonau E, et al. Measuring procedures to determine the Cobb angle in idiopathic scoliosis: a systematic review. Eur Spine J. 2013;22(11):2360-71.

40. Mannion AF, Knecht K, Balaban G, Dvorak J, Grob D. A new skin-surface device for measuring the curvature and global and segmental ranges of motion of the spine: reliability of measurements and comparison with data reviewed from the literature. European Spine Journal. 2004;13(2):122-36.

41. Livanelioglu A, Kaya F, Nabiyev V, Demirkiran G, Fırat T. The validity and reliability of “Spinal Mouse” assessment of spinal curvatures in the frontal plane in pediatric adolescent idiopathic thoraco-lumbar curves. European Spine Journal. 2016;25(2):476-82.

42. Lenke LG, Betz RR, Harms J, Bridwell KH, Clements DH, Lowe TG, et al. Adolescent idiopathic scoliosis: a new classification to determine extent of spinal arthrodesis. JBJS. 2001;83(8):1169-81.

43. Lenke LG, Edwards CC, Bridwell KH. The Lenke classification of adolescent idiopathic scoliosis: how it organizes curve patterns as a template to perform selective fusions of the spine. Spine. 2003;28(20S):S199-S207.

44. Sanders JO, Polly Jr DW, Cats-Baril W, Jones J, Lenke LG, O’Brien MF, et al. Analysis of patient and parent assessment of deformity in idiopathic scoliosis using the Walter Reed Visual Assessment Scale. Spine. 2003;28(18):2158-63.

45. Asher M, Lai SM, Burton D, Manna B. The reliability and concurrent validity of the scoliosis research society-22 patient questionnaire for idiopathic scoliosis. Spine. 2003;28(1):63-9.

46. Coelho DM, Bonagamba GH, Oliveira AS. Scoliometer measurements of patients with idiopathic scoliosis. Brazilian journal of physical therapy. 2013;17(2):179-84.
