# Supplementary material for: The effectiveness of two different exercise approaches in adolescent idiopathic scoliosis: A single-blind, randomized-controlled trial
Source: PLoS One. 2021 Apr 15;16(4):e0249492. doi: 10.1371/journal.pone.0249492 (PMC8049223; doi:10.1371/journal.pone.0249492)
Supplement: S2 File — (DOC) [file pone.0249492.s004.doc]

**T.C**

**AHİ EVRAN ÜNİVERSİTESİ**

**KLİNİK ARAŞTIRMALAR ETİK KURULU**

**(Çalışma Protokolü)**

Belge Tarihi : 18.09.2019

Versiyon No : 2

**Araştırma Projesinin Adı:** “Adölesan İdiyopatik Skolyozda İki Farklı Egzersiz Yönteminin Skolyoz Şiddeti, Yürüyüş, Fiziksel, Fonksiyonel Parametreler ve Yaşam Kalitesi Üzerine Etkisinin İncelenmesi”

**Koordinatör:** Mehmet YETİŞ

**Araştırmacılar:** Hikmet KOCAMAN

Nilgün BEK

Mehmet Hanifi KAYA

Buket BÜYÜKTURAN

Öznur BÜYÜKTURAN

**Projenin Uygulanacağı Yer:** Kırşehir Ahi Evran Üniversitesi Fizik Tedavi ve Rehabilitasyon Yüksekokulu

**Araştırmanın Niteliği:** Randomize Kontrollü Tek Kör Çalışma

**Tahmini Araştırma süresi:** 6 ay

**Giriş ve Amaç**

Adolesan idiyopatik skolyoz (AIS), omurganın üç boyutlu deformitesi ile karakterize, etiyolojisi bilinmeyen ilerleyici bir büyüme hastalığıdır (radyografilerde frontal translasyon, sagital modifikasyon ve skolyotik eğrinin konkav tarafında spinöz çıkıntıların torsiyonu).

AIS'li hastalarda, eğrinin ilerlemesi ile birlikte yaygın olarak kas dengesizliği, fonksiyonel kısıtlılıklar, değişen postür, yürüş deviasyonları, omurganın esnekliğinde azalma, sırt ağrısı, negatif fiziko-sosyal, vücut imajı etkileri ve ciddi vakalarda pulmoner semptomlar gibi problemler görülebilmektedir. Skolyozun şekli ve açısına bağlı olarak gövde ve pelvis simetrisi etkilenir. Skolyozun şekline ve Cobb açısına bağlı olarak ağırlık dağılım pozisyonu değişir. Bu komplikasyonların ve daha fazlasının üstesinden gelmek için, AIS için egzersiz, korseleme, alçılama, traksiyon, biofeedback, cerrahi ve basit gözlem gibi deformitenin ilerlemesini önlemek, düzeltmek veya durdurmak için çeşitli tedavi yaklaşımları önerilmiştir. Orta Avrupa'da fizyoterapi ve korse konservatif tedavi yöntemleri olarak kabul edilmektedir.

Literatürde egzersizler ilerlemeyi azaltmak, omurga ve göğüs kafesi esnekliğini artırmak, kas kuvvetlerini ve esnekliği iyileştirmek, postural davranışı düzeltmek, nöro-motor kontrol ve omurga stabilitesini artırmak için önerilmektedir.

Genel olarak, postural eğitim, omurga kasları için germe ve güçlendirme egzersizleri, solunum egzersizleri gibi egzersizleri içeren geleneksel egzersizler, skolyoz için uzun yıllardır kullanılmaktadır.

Geleneksel egzersizler dışında, Schroth, Side Shift, Dobomed vb. yöntemler dahil olmak üzere çeşitli egzersiz konseptleri vardır. Schroth egzersizleri, eğriyi, fonksiyonu, postürü, vücut imajını ve ağrıyı iyileştirmeyi amaçlayan asimetrik, skolyoza özgü postüral egzersizlerdir. Schroth egzersizleri sırt, karın ve bacak kaslarının güç ve dayanıklılık eğitimini hedefler. Ayrıca Schroth egzersizlerinin amaçlarından biri de düzeltici hareketleri giderek daha az geribildirimle tekrarlayarak postürün motor kontrolünü iyileştirmektir. Schroth egzersizleri en çok çalışılan skolyoz egzersizleridir ancak Schroth egzersizleriyle ilgili sınırlı randomize kontrollü çalışma vardır.

Son zamanlarda, core stabilizasyon (CS) egzersizleri, Pilates gibi genel fizyoterapötik egzersizler, idiyopatik skolyozun konservatif tedavisinde kullanılmaktadır. CS egzersizleri, nöromüsküler kontrolü, gövde stabilizasyon kaslarının gücünü, omurga çevresindeki postüral kasların dayanıklılığını, pelvis ve omurga arasındaki dengeyi artırarak postüral kontrolü ve fonksiyonel stabiliteyi iyileştiren terapi teknikleri olarak tanımlanmaktadır. Bununla birlikte, sınırlı sayıda çalışmada, CS egzersizlerinin AIS'li hastalar üzerindeki etkisini belirtilmiştir. Bir çalışmada CS egzersizlerinin, AİS'nin konservatif rehabilitasyonunda geleneksel egzersizlere göre ağrıyı ve rotasyonel deformiteyi azaltmada daha etkili olduğu bulunmuştur.

Yakın zamanda yapılan bir sistematik inceleme, terapötik egzersizin semptomları, Cobb açısını, gövde rotasyonunu, kraniovertebral açıyı ve vücut asimetrilerini azaltmada ve AIS'li hastaların kassal enduransını, pulmoner fonksiyonunu ve fonksiyonel kapasitesini iyileştirmede etkili olduğunu göstermiştir. Düzeltici, terapötik egzersizlerin fonksiyonları iyileştirerek ve semptomları azaltarak, çeşitli açıları ve vücut asimetrilerini azaltarak olumlu etkileri olduğu görülmektedir. Bununla birlikte, bu sonuçları doğrulamak ve en iyi terapötik egzersiz müdahalesini tespit etmek için daha iyi metodolojik kaliteye sahip ileri çalışmalara ihtiyaç vardır. Ayrıca klinik uygulamada en etkili egzersizi seçmek için farklı egzersiz yöntemlerine yönelik randomize kontrollü çalışmalara ihtiyaç vardır.

Literatürde AIS'li hastalarda Schroth yöntemi ve CS egzersizlerinin karşılaştırıldığı araştırmaya rastlanmamıştır. Ayrıca Schroth yönteminin periferik kas kuvvetleri üzerindeki etkilerini inceleyen bir araştırma da bulunamamıştır. Bu çalışmada, geleneksel egzersizlere ek olarak uygulanan Schroth ve CS egzersizlerinin AIS ‘li hastalarda Cobb açısı, gövde rotasyonu, periferik kas kuvvetleri, omurga hareketliliği, kozmetik deformite, pedobarografik yürüyüş parametreleri ve sağlıkla ilişkili yaşam kalitesi üzerine etkilerinin araştırılması amaçlanmıştır.

**Gereç ve Yöntem**

Bu çalışmada Adölesan İdiyopatik Skolyoz’da (AİS), iki farklı egzersiz yönteminin, skolyoz şiddeti, yürüyüş, fiziksel ve fonksiyonel parametreler ve yaşam kalitesi üzerine etkileri incelenecektir. Çalışmaya güç analizi sonucuna göre gerekli olan 28 AİS'li birey dahil edilip eşli randomizyon ile iki gruba ayrılıp bir gruba konvansiyonel egzersizlere ilave olarak core stabilizasyon egzersizleri uygulanırken, diğer gruba konvansiyonel egzersizlere ilave olarak skolyoza özel 3 boyutlu egzersizlerden Schroth egzersizleri uygulanacaktır. Çalışma kapsamında Lenke sınıflamasına göre eğri tipi tip 1 olan adolesanların demografik bilgileri alındıktan sonra, Risser'e göre kemik maturasyon seviyeleri, eğriyi içeren omurga bölgeleri kaydedilecektir. 10 haftalık Schroth ve core stabilizasyon egzersiz programını içeren tedavi öncesinde ve tedaviyi takiben, ön-arka röntgen grafisi yardımıyla eğrilerine ait Cobb açıları, öne eğilme testinde skolyometre ile gövde rotasyonları, Biodex Sistem 4 Pro® izokinetik cihazı ile üst ekstremite patern ve alt ekstremite izokinetik kas kuvvetleri, Spinal mouse ile spinal mobiliteleri, Walter Reed Visuel Assessment Scale (WRVAS)'a göre kozmetik deformite algıları, SRS-22 anketine göre yaşam kaliteleri, DIASU Dijital Analiz Sistemi® ve Milletrix yazılımı (DIASU, İtalya) ile pedobarografik yürüyüş parametreleri değerlendirilecektir. Çalışmaya Ahi Evran Üniversitesi Eğitim ve Araştırma Hastanesi Ortopedi ve Travmatoloji Polikliniğine başvuran bireyler dahil edilecektir. Bu bireyler dahil edilme kriterlerini sağlamaları durumunda Ahi Evran Üniversitesi Fizik Tedavi ve Rehabilitasyon Yüksekokuluna yönlendirileceklerdir. G*Power version 3.0.10. ile α=0.05 istatistiksel anlamlılık düzeyinde, Cohen's d katsayısı IrI: 0.30 etki genişliğinde β:0.80 güç elde edebilmek için gerekli olan 28 birey alınıp, bu olgular eşli randomizasyon sistemi ile yaş, eğrilik derecesi ve Risser bulgusu açısından randomize olarak 2 gruba ayrılacaktır. Çalışmamıza başlamadan önce Ahi Evran Üniversitesi klinik araştırmalar etik kurulundan onay alınacaktır ve çalışma Helsinki deklerasyonuna uygun yapılacaktır. Araştırmaya katılan tüm hasta ve ailelerine çalışmanın içeriği ve uygulanma biçimi, katılımcı hakları, kullanılacak olan anketler hakkında sözlü ve yazılı olarak bilgi verilerek onamları alınacaktır.

**DAHİL OLMA ve DIŞLAMA KRİTERLERİ**

Dahil Edilme Kriterleri;

1. 10-18 yaşları arasında olup AİS tanısı almış olmak
2. Korse kullanmamak,
3. Cobb açısının 10º-30º arasında olması,
4. Lenke tip 1 eğriliğe sahip olmak
5. Uygulanacak programa devam edebilmesi,
6. Kosta kırığı, atelektazi, astım gibi pulmoner veya göğüs kafesi ile ilgili hastalığı olmaması
7. Herhangi bir nörolojik, psikiyatrik hastalığının olmaması ve ilaç kullanımını gerektiren kronik hastalığının bulunmaması,
8. Ebeveynlerinin çocuğun çalışmaya katılmasına izin vermesidir.

Dahil Edilmeme Kriterleri;

1. Hastanın egzersiz yapması için herhangi bir kontraendikasyonunun olması,
2. Daha önce skolyoz tedavisi almış olması veya omurga cerrahisi geçirmiş olması,
3. Korse kullanması
4. Herhangi bir mental probleminin bulunması,
5. Skolyozun idiopatik olmayıp farklı nedenlerle ortaya çıkmış olması (nörolojik, konjenital vb…),
6. Skolyoz dışında yürüyüşü etkileyecek herhangi bir problemin olması

**Veri Toplama Araçları ve Özellikleri**

1) Sosyodemografik Değerlendirme

- Yaş, boy, kilo, vücut kitle indeksi, özgeçmiş, soygeçmiş, dominant tarafı, Risser bulgusu

2) Eğriliğin derecesinin Cobb açısı

3) Spinal mouse ile spinal mobilitenin ölçülmesi

4) Gövde rotasyon derecesinin belirlenmesinde skolyometre ile ölçülmesi

5) Üst ekstremite patern (fleksiyon-abduksiyon-eksternal rotasyon / ekstansiyon-adduksiyon-internal rotasyon) kas kuvveti ve alt ekstremitede quadriceps, hamstring kas kuvvetlerinin saptanmasında izokinetik kuvvet ölçümleri

6) Walter Reed Görsel Değerlendirme Skalası (WRVAS) ile kozmetik deformitelerini algılamalarının değerlendirilmesi

7) Scoliosis Research Society-22 anketi ile yaşam kalitesinin değerlendirilmesi

8) Yürüyüş parametrelerinin değerlendirilmesi DIASU Dijital Analiz Sistemi® ve Milletrix yazılımı (DIASU, İtalya) ile gerçekleştirilecektir.

**Sosyo-Demografik Değerlendirme:**

Bireylerin yaşı, vücut ağırlığı ve boyu, anemnezi (soygeçmiş, özgeçmiş), medikal hikayeleri, menarş yaşı, dominant el ve ayakları, egzersiz alışkanlıkları (önceden yaptığı sporlar) ve süreleri sorgulanıp kaydedilecektir.

**Risser bulgusu (İlliak apofiz):** İlk olarak 1958 yılında Joseph C. Risser tarafından tanımlanmıştır. Risser, iliak apofizin ossifikasyon durumunun spinal iskeletin gelişim durumu ile ilişkili olduğunu gözlemlemiş ve AİS’nin tedavisinde kritik bir bilgi olduğunu belirtmiştir. İliak apofizin anterolateralden posteromediale doğru ossifikasyonuna göre 0-5 arasında (0: kemik füzyonu hiç başlamamış; 5: kemik füzyonu tamamlanmış) derecelendirme yapılarak değerlendirilir. Risser bulgusu kemik gelişim yaşını, büyüme hızını ve skolyoz için risk derecesini belirlemede kullanılmaktadır. Risser derecesi, eğriliğin progresyonu ile doğrudan ilişkilidir ve iskelet matürasyonu tamamlandıkça skolyozun ilerleme riski azalmaktadır. Çalışmamızda kemik matürasyon derecesini belirlemek amacı ile kullanılacaktır.

**Cobb Açısı:** Skolyozda koronal düzlemdeki deformitenin değerlendirilmesini sağlar. Ön arka yönde, ayakta çekilen, tüm omurgayı içeren omurga röntgeninden skolyozun lateral fleksiyon açısının ölçümü yapılır. Eğrilik derecesinin belirlenmesinde Cobb metodu altın standart ölçüm yöntemi olarak kabul edilir. Cobb açısı üç boyutlu bir deformitenin sadece bir düzlemini tanımlamaktadır, ancak eğriliğin ilerleyişi hakkında bilgi vermektedir. Cobb açısı başlangıçta ve 10 haftalık tedavi sonrası olmak üzere toplam 2 kez çektirilen radyografi üzerinden aynı vertebral son plaklar temel alınarak ölçülecektir. Çalışmamızda, 10 haftalık tedavinin etkisini değerlendirmek için Cobb açısına göre değişim derecesine bakılacaktır.

**Lenke Sınıflaması:** Bu sınıflamanın altı adet eğrilik tipi, lomber omurga işaretleyicisi, torakal omurga işaretleyicisi olarak üç bileşeni vardır. Öncelikle eğriliğin yeri proksimal torasik, torasik, torakolomber ya da lomber olarak belirlenir. Daha sonra en büyük Cobb açısı olan deformite majör eğrilik, diğerleri de minör eğrilik olarak adlandırılır. Esnekliği olmayan eğriliklere yapısal eğrilik denir. Lenke Tip I’de ana torakalde majör eğrilik mevcuttur. Proksimal torakal ve lumbotorakaldeki eğrilikler minördür ve yapısal değildir. Lenke Tip II eğriliklerde çift torakal eğrilik mevcuttur. Proksimal torakaldeki ve ana torakaldeki eğrilikler yapısal, lumbotorakaldeki eğrilik yapısal değildir. Lenke Tip III’de ana torakalde temel, torakolomberde yapısal eğrilik mevcuttur. Üçlü eğrilikler Lenke Tip IV’dür. Lenke Tip V’de ana eğrilik torakolomber bileşkededir. Proksimal torakaldeki ve ana torakaldeki eğrilikler yapısal değildir. Lenke Tip VI’da ana torakaldeki ve torakolomber/lomber bölgedeki eğrilik yapısaldır ve ana torakaldeki eğrilikten daha büyüktür. Çalışmamızda Lenke Tip I eğriliği olan bireyler alınacaktır.

**Kozmetik deformite algılaması:** Bireylerin kozmetik deformitelerini algılamalarının değerlendirilmesi ve tedavinin vücut kozmetik deformitesini iyileştirmedeki etkinliğini değerlendirmek için, Walter Reed Görsel Değerlendirme Skalası (WRVAS) kullanılacaktır. WRVAS, vücut eğriliği, kaburganın belirginliği, bel çıkıntısının belirginliği, baş-kaburga-pelvis pozisyonel ilişkisi, baş-pelvis ilişkisi, omuz seviyesi ve skapula rotasyonunu içeren 5 farklı figürün gösterildiği 7 parametreye ayrılmaktadır. Her bir parametre ise 1’den 5’e doğru şiddeti artan şekilde skorlanır. Kişi kendi vücuduna uygun olanı 1-5 arası işaretler. Kişinin duruş algısına odaklanarak eğri şiddetini skorlar.

**Sağlıkla ilgili yaşam kalitesinin değerlendirilmesi:** Scoliosis Research Society-22 anketi (SRS-22) spinal deformiteli hastalar için özel olarak tasarlanmış sağlıkla ilgili yaşam kalitesi anketidir. SRS-22 fonksiyon, ağrı, mental sağlık, vücut imajı ve tedaviden tatmin olarak 5 ana başlık içermektedir. Her madde için puan sıfır (en kötü) ile beş (en iyi) arasında değişmektedir. Tedavi öncesi ve sonrasında yaşam kalitesini değerlendirmek için kullanılacaktır.

**Gövde Rotasyon Derecesi:** Skolyoza bağlı gelişen horizontal düzlemdeki değişimler için gövde rotasyon asimetrisinin skolyometre adı verilen özel bir inklinometre ile ölçülmesi klinikte sık kullanılan bir yöntemdir. Skolyometrenin kişilerarası güvenirliği "mükemmel" olarak bulunurken, ölçümlerarası güvenirliğinin "çok iyi" olduğu belirtilmiştir. Gövde rotasyonu fizyoterapist tarafından skolyometre ile öne eğilme testinde apeks vertebranın rotasyon açısı ölçülerek derece cinsinden kaydedilecektir.

**Kas Kuvveti**: Çalışmaya dahil edilecek bireylerin her iki taraf üst ekstremite (fleksiyon-abduksiyon-eksternal rotasyon-ekstansiyon-adduksiyon-internal rotasyon) patern kas kuvveti ve alt ekstremitede quadriceps, hamstring kas kuvveti Biodex Sistem 4 Pro® (Biodex Corp, Shirley, NY) cihazı kullanılarak hem tedavi öncesi hem de tedavi sonrası değerlendirilecektir. Maksimum izokinetik kas kuvveti 60˚/sn hızda 5 tekrar, 120˚/sn 10 tekrar olarak ölçülecektir.

**Spinal Mobilite Değerlendirmesi:** Spinal mobilitenin değerlendirilmesi, Spinal Mouse (SM) adı verilen taşınabilir, bilgisayar destekli elektromekanik cihaz (the Spinal Mouse System, Idiag, Fehraltorf, Switzerland) ile yapılacaktır. SM, frontal ve sagital düzlemlerde spinal açıları ve eğrilikleri değerlendirebilen, harici, invaziv olmayan bir ölçüm cihazıdır. SM'nin AİS’de klinikte araştırma ve hasta takibi için, yan etkisi olmayan, güvenilir, hızlı ve kullanımı kolay bir ölçüm yöntemi olarak kullanılabileceği bildirilmiştir. Ölçümler, servikal 7. vertebranın spinöz çıkıntısı ile anal kıvrımın tepesi (yaklaşık olarak sakral 3. vertebra seviyesi) arasında yapılacaktır. Frontal düzlemde maksimum sağ-sol lateral fleksiyon dereceleri ile sagittal düzlemde maksimum fleksiyon-ekstansiyon dereceleri ölçülerek kaydedilecektir.

**Pedobarografik Yürüyüş Değerlendirmesi**: Özel bir platforma yerleştirilen elektronik sensörler ve sensörlerden gelen bilginin analizini gerçekleştiren bilgisayar sistemi ile yürüyüşün belirli evrelerinde ayağın durumu ve ayağa binen yüklerin ölçümleri gerçekleştirilebilmektedir. Çalışmamızda bireylerin statik, dinamik pedobarografik yürüyüş analizleri 3x1 metre basınç sensörlü yürüyüş platformuna sahip DIASU Dijital Analiz Sistemi® ve Milletrix yazılımı (DIASU, İtalya) ile gerçekleştirilecektir. Statik ölçümde bireylerin kendini rahat hissettiği pozisyonda durması istenip ve statik koşullardaki temas yüzdesi (%), maksimal basınç (kg/cm^2^) ile sağ-sol ayak için ayrı ayrı olmak üzere; temas alanı (cm^2^), temas yüzeyi (%) değerleri kaydedilecektir.

Dinamik ölçümlerde, bireylerin 3 m’ lik içine basınç ölçer sensorleri gömülmüş yürüme yüzeyi üzerinde, normal yürüme hızında üç tekrar olacak şekilde yürümeleri istenecektir. Bireylerden elde edilen verilerin ortalamaları alınarak, dinamik koşullarda, ön-arka ayağa etkiyen yükler (kg), ortalama basınçlar (kg/cm^2^) sağ-sol ayak için ayrı ayrı olmak üzere; temas alanı (cm^2^), temas yüzeyi (%) kaydedilecektir.

**Çalışmada Uygulanacak Konvansiyonel Egzersizler**

Sırt ve omuz kuşak kaslarının güçlendirilmesi, eğrinin içbükey tarafı için germe egzersizleri, postüral eğitim, omurga için esneklik egzersizleri ve nefes egzersizleri yapılacaktır.

**Çalışmada Uygulanacak Schroth Egzersizleri**

Çalışmamıza katılmayı kabul eden bireylerden Schroth grubuna dahil olanlara, Uluslararası Schroth 3 Boyutlu Skolyoz Terapisi (ISST) eğitimi almış deneyimli bir fizyoterapist tarafından haftada 3 gün, klasik egzersizlerle birlikte her seansı yaklaşık 90 dakika süren, 10 haftalık Schroth egzersiz programı uygulanacaktır. Schroth yönteminde egzersizler eğriliğin yapısına ve kişiye özel olarak planlanarak uygulanacaktır. İlk olarak primer eğriliklerinin, varsa sekonder eğriliklerinin meydana getirdiği postural deviasyonlar ve nefes bölgelerinin tanımlanması yapılıp 3 boyutlu düzeltici solunum egzersizinin öğretilmesi ile egzersiz programına başlanacaktır. Egzersizlerin yapılmasında duvar barı, ayna, egzersiz matı, 3 adet pirinç torbası, sünger yastık, sandalye, iki adet uzun sopa gibi yardımcı materyaller kullanılacaktır. Egzersizler basit sırtüstü ve yan yatış pozisyonlarında başlandıktan sonra gittikçe zorlukluğu artan (oturma pozisyonu, ayakta, yürüme) pozisyonlarda devam edilecektir. Egzersizler, 7-10 tekrardan 10-15 tekrara aşamalı olarak ilerletilecektir. 10 hafta boyunca uygulanacak Schroth egzersizleri Tablo 1’de gösterilmiştir.

**Tablo 1.** Çalışmada uygulanacak Schroth egzersizleri

| **Başlangıç Seviyesi** | **Orta Seviye** | **İleri Seviye** |
| --- | --- | --- |
| 3 boyutlu düzeltici solunum egzersizi | 3 boyutlu düzeltici solunum egzersizi | 3 boyutlu düzeltici solunum egzersizi |
| Sırtüstü shoulder counter-traction | Oturma pozisyonunda Shoulder counter-traction | Oturma pozisyonunda Shoulder counter-traction |
| Yüzüstü shoulder counter-traction | Chest twister | Chest twister |
| Yan yatışta shoulder counter-traction | Dizüstü pozisyonunda muscle cylinder | Dizüstü pozisyonunda muscle cylinder |
| Sırtüstü muscle cylinder | Big bow | Big bow |
| Yan yatışta muscle cylinder | Shoulder counter-traction between two poles | Shoulder counter-traction between two poles |
| Oturma pozisyonunda muscle cylinder | Schroth gait | Schroth gait |
| Chest twister | Removing the stool | Removing the stool |

*Düzeltici/Rotasyonel Solunum:* 3 boyutlu deformitede kompresyon altında kollabe olmuş konkav alanlara seçici nefes alınarak, kostal aralıkların uzatılması ve yumuşak dokuların mobilizasyonu sağlanmaktadır. Spontan solunumdan farkı, bireyin komprese olmuş alanlara odaklanarak inspirasyonu lokal olarak o bölgelere yapmasıdır. İlk başlarda bunu sağlamak için el teması ve sözel bildirimler kullanılarak bireyin alınan havayı doğru alana yönlendirmesi sağlanır. Daha sonra rotasyonel solunum komponenti tüm egzersizlerle kombine edilerek, postural düzeltmenin rotasyonel solunum egzersizleri ile desteklenmesi sağlanmaktadır.

*Shoulder counter traction*: Torakal eğriliği düzeltmek amacıyla omuzun karşı traksiyonuyla beraber defleksiyon (omurganın lateral deviasyonunu orta hatta yaklaştırma/ düzetme) ve derotasyonun sağlanmaya çalışıldığı bu egzersiz, çalışmamızda yüzüstü, sırtüstü, yan yatış ve oturma pozisyonda uygulanmıştır. Rotasyonel solunumla beraber nefes verme sırasında torakal konveksite orta hatta doğru itilirken, omurgadaki rotasyonunun tersine rotasyon yapılmaya çalışılır. Aynı zamanda konveks taraftaki omuzun karşı traksiyonuyla maksimum düzeltme sağlanmaya çalışılır.

*Muscle Cylinder (Kas silindiri):* Bu egzersizde torakal eğriliğin defleksiyonu, topuğun kaudale itilmesiyle lumbar eğriliğin defleksiyonu ve quadratus lumborum kasının egzentrik çalıştırılması amaçlanmaktadır. Yan yatışta, yarım dizüstü pozisyonda ve ayakta yapılabilmektedir. Yan yatışta birey torakal konkav taraf üzerine yatırılır, torakal konkavitedeki kompresyonun azaltılması için konkav taraftaki kol başın üzerine uzatılır ve lumbar eğriliğin düzeltilmesi amacıyla lumbar bölgeye pirinç torbası yerleştirilir. Konveks taraftaki omuz retraksiyona alınır ve üstte kalan el pelvis üzerine yerleştirilip alt ekstremite kaudale uzatılırken ayak yerden bir miktar kaldırılarak lomber konkavitenin düzeltilmesine çalışılır.

*Removing the stool:* Omurganın elongasyonu ile birlikte sırt ve çevre kaslarının kuvvetlendirilmesinin sağlanmaya çalışıldığı bu egzersizde birey barın önünde bacakları bağdaş kurma pozisyonunda bir tabure üzerinde oturur ve kollarıyla bardan tutunur. Düzeltici/rotasyonel solunum paterni ile birlikte nefes verirken temel düzeltmeleri yapar ve oturduğu tabure çekilerek pozisyonunu 3-4 nefes alıp verme süresinde muhafaza etmesi istenir.

*Big bow:* Omurganın uzatılması ve elongasyonunun sağlanmaya çalışıldığı bu egzersizde, birey barın önünde, bardan tutar ve hamstring kaslarının gerginliğini azaltmak için dizlerini hafif büker. Bu pozisyondayken eğriliğin tipine göre torakal, lumbar ve pelvik düzeltmeler yapılır. Düzeltici/rotasyonel solunum paterni ile birlikte nefes verirken kolları ekstansiyona alınıp torakal fleksiyon yapılır. Bu egzersizde torakal fleksiyon sırasında torakal bölgedeki kifozun ve lumbar lordozun korunmasına dikkat edilmelidir.

*Between two poles (İki sopa arası):* Bu egzersizde sopalar kullanılarak omuz kuşağının karşı-traksiyonu ile ayakta durma pozisyonundaki temel düzeltmelerin yapılması hedeflenmektedir. Sopalar yere dik, konveks taraf ön kol sopaya bitişik konkav taraf kol düz bir şekilde baş üstü pozisyonda pozisyonlanır. Egzersiz pozisyonunu aldıktan sonra pelvik düzeltmeler (shift, tilt, rotasyon yönünde) ve doğru ağırlık aktarımı ile başlanır, aksiyal elongasyon yapılır ve ardından düzeltici/rotasyonel solunum ile eğriliğin tipine göre hastadan temel düzeltme hareketleri (defleksiyon, derotasyon) istenir.

*Chest Twister:* Bu egzersizde fikse edilmiş pelvis ile torakal bölgede defleksiyon ve derotasyon sağlanmaya çalışılır. Temel düzeltmeler (kaydırma, rotasyon) over-correction (aşırı düzeltme) şeklinde uygulanır. Birey yüzü bara dönük şekilde sandalyede otururken kollarla diagonal pozisyonda bardan tutar. Gövdesini torakal konkav tarafa kaydırır. Bu arada, lumbar konkavitenin defleksiyonu ve pelvik asimetrinin düzeltilmesi için lomber konkav taraf pelvis sandalye dışında pozisyonlanır. Düzeltici/rotasyonel solunumla birlikte temel düzeltme hareketleri tekrarlanır.

*Schroth gait (Schroth yürüyüşü):* Dinamik koşullarda düzeltmelerin devamlılığı ve stabilizasyonunun amaçlandığı bu egzersizde, egzersiz pozisyonu aldıktan sonra üç düzlemdeki düzeltmeler yapılır ve düzeltici/rotasyonel solunum paterni ile birlikte nefes verirken parmak ucunda yükselip maksimum aksiyal elongasyon yapması istenir. Öne doğru atılan her adımda tekrarlanır.

**Çalışmada Uygulanacak Core Stabilizasyon Egzersizleri**

Literatürde skolyozda uygulanan core stabilizasyon egzersizleri referans alınarak belirlediğimiz Core Stabilizasyon egzersiz programı kolaydan zora doğru olacak şekilde 3 aşamalı olarak uygulanacaktır. İlk olarak bireylere trasversus abdominus kasını nasıl aktive edecekleri öğretildi. Bu doğrultuda sırtüstü yatışta çengel pozisyonunda spina iliaka anterior superiorların anteromedialine yerleştirdikleri elleriyle transversus abdominus kasının kontraksiyonunu palpe edip hissedebilmeleri sağlanarak, transversus abdominus kasının aktivasyonu gösterilecektir. Daha sonra hastalardan transversus abdominus ve multifidus kaslarının kontraksiyonunu, farklı pozisyonlarda ve vücudun diğer kaslarının da aktivasyonunu içeren egzersizler sırasında korumaları istenecektir. Tüm egzersizler sırasında solunum kontrolünün önemi vurgulanarak, özellikle hareketin zorlu komponenti sırasında valsalva manevrasına yol açmaması için nefes vererek hareketleri yapmaları istenecektir. Her hareket önce fizyoterapist tarafından gösterildi ve hastaların da hareketleri doğru bir şekilde yapmaları için egzersizler sırasında sözlü veya taktil uyaranlar ile hataları düzeltilecektir. Her bir eğitim seansı, 10 dakikalık bir ısınma programı ile başlayıp 10 dakikalık bir soğuma programı ile sona eren klasik egzersizlerle birlikte toplamda 90 dakika süren bir eğitim programından oluşacaktır.

Egzersizlerin progresyonu hastaların yapabilirliğine göre kişiye özel ilerletilecektir. Egzersizler 7-10 tekrardan 10-15 tekrara ilerleyecek şekilde tasarlandı. Ayrıca egzersizler pozisyon değişiklikleri, vücut ağırlığının kullanımı, Thera band (Theraband Elastic Band Hygienic Corporation, Akron, Ohio) ve egzersiz topu kullanımı gibi aşama aşama yöntemlerle zorlaştırılacaktır.

Üç aşama olarak planlanan programda ilk 3 hafta başlangıç seviyesi, sonraki 3 hafta orta seviye ve son 4 hafta da ileri seviye olarak belirlendi. Hastalar başladıkları aşamayı tamamlayıp diğer aşamaya geçtiklerinde egzersizlerde yine az tekrar sayısından çok tekrar sayısına doğru ilerlenecektir. Bir sonraki seviyede başarılamayan hareket olduğunda bir süre daha aynı programdaki harekete devam edilip, daha sonra başarılamayan hareket yapılacaktır. Hastalara uygulanacak core stabilizasyon egzersizleri Tablo 2’de gösterilmektedir.

**Tablo 2. Çalışmada uygulanacak Core Stabilizasyon egzersizleri**

| **Başlangıç Seviyesi** | **Orta Seviye** | **İleri Seviye** |
| --- | --- | --- |
| Sırtüstü kanca pozisyonunda TrA ve ML kaslarının aktivasyonunu öğrenme | Isınma Egzersizleri | Isınma Egzersizleri |
| Egzersizler sırasında nötral lumbopelvik kontrolün devamının eğitimi | Sarı Theraband ile düz bacak kaldırma | Kırmızı Theraband ile düz bacak kaldırma |
| Isınma Egzersizleri | Sırtüstü çapraz kol-bacak kaldırma | Abdominal Curl |
| Sırtüstü düz bacak kaldırma | Sırtüstü bisiklet egzersizi | Supine Bridge Ball Rolls |
| Sırtüstü kalça fleksiyonu | Tek ayak üzerinde köprü | Dizler fleksiyonda, ayaklar top üzerinde iken köprü egzersizi |
| Sırtüstü omuz fleksiyonu | Dizler ekstansiyonda, ayaklar top üzerinde iken köprü egzersizi | Yüzüstü köprü |
| Sırtüstü köprü | Sırtüstü ayaklarla top yuvarlama | Dizler ekstansiyonda yan köprü egzersizi |
| Midye egzersizi | Dizler fleksiyonda yan köprü egzersizi | Side Bridge |
| Kalça abduksiyonu | Sarı Theraband ile kalça abduksiyonu | Kırmızı Theraband ile kalça abduksiyonu |
| Kedi-deve egzersizi | Sarı Theraband ile midye egzersizi | Kırmızı Theraband ile midye egzersizi |
| Superman (kol) | Çapraz kol bacak superman egzersizi | Kedi-deve egzersizi |
| Superman (bacak) | Kedi-deve egzersizi | Çapraz kol bacak superman egzersizi |
| Top üzerinde otururken Core stabilizasyon ile pelvik tilt | Top üstünde otururken kalça fleksiyonu | Top üstünde otururken çapraz kol-kalça fleksiyonu |
| Top üstünde otururken üst ektremite PNF egzersizleri | Top üstünde otururken sarı Theraband ile üst ekstremite PNF egzersizleri | Top üstünde otururken kırmızı Theraband ile üst ekstremite PNF egzersizleri |
| Ayakta üst ektremite PNF egzersizleri | Ayakta sarı Theraband ile üst ekstremite PNF egzersizleri | Ayakta kırmızı Theraband ile üst ekstremite PNF egzersizleri |
| Soğuma Egzersizleri | Soğuma Egzersizleri | Soğuma Egzersizleri |

**Kaynaklar**

1. Weinstein SL, Dolan LA, Cheng JCY, Danielsson A, Morcuende JA. Adolescent idiopathic scoliosis. The Lancet. 2008;371(9623):1527-37.

2. Tones M, Moss N, Polly Jr DW. A review of quality of life and psychosocial issues in scoliosis. Spine. 2006;31(26):3027-38.

3. Burgoyne W, Fairbank J. The management of scoliosis. Current Paediatrics. 2001;11(5):323-31.

4. Goldstein L, Waugh T. Classification and terminology of scoliosis. Clinical Orthopaedics and Related Research®. 1973;93:10-22.

5. James JIP. Idiopathic scoliosis: the prognosis, diagnosis, and operative indications related to curve patterns and the age at onset. The Journal of bone and joint surgery British volume. 1954;36(1):36-49.

6. Altaf F, Gibson A, Dannawi Z, Noordeen H. Adolescent idiopathic scoliosis. Bmj. 2013;346:f2508.

7. Lonstein J, Carlson J. The prediction of curve progression in untreated idiopathic scoliosis. J Bone Jt Surg. 1984:1061-71.

8. Sanders JO, Browne RH, Cooney TE, Finegold DN, McConnell SJ, Margraf SA. Correlates of the peak height velocity in girls with idiopathic scoliosis. Spine. 2006;31(20):2289-95.

9. Negrini S, Aulisa AG, Aulisa L, Circo AB, de Mauroy JC, Durmala J, et al. 2011 SOSORT guidelines: orthopaedic and rehabilitation treatment of idiopathic scoliosis during growth. Scoliosis. 2012;7(1):3.

10. Martínez-Llorens J, Ramirez M, Colomina M, Bagó J, Molina A, Cáceres E, et al. Muscle dysfunction and exercise limitation in adolescent idiopathic scoliosis. European Respiratory Journal. 2010;36(2):393-400.

11. Enneking W, HARRINGTON P. Pathological changes in scoliosis. JBJS. 1969;51(1):165-84.

12. Shea KG, Ford T, Bloebaum RD, D'astous J, King H. A comparison of the microarchitectural bone adaptations of the concave and convex thoracic spinal facets in idiopathic scoliosis. JBJS. 2004;86(5):1000-6.

13. Fidler M, Jowett R. Muscle imbalance in the aetiology of scoliosis. The Journal of bone and joint surgery British volume. 1976;58(2):200-1.

14. Zapata KA, Wang-Price SS, Sucato DJ, Dempsey-Robertson M. Ultrasonographic measurements of paraspinal muscle thickness in adolescent idiopathic scoliosis: a comparison and reliability study. Pediatric Physical Therapy. 2015;27(2):119-25.

15. Stetkarova I, Zamecnik J, Bocek V, Vasko P, Brabec K, Krbec M. Electrophysiological and histological changes of paraspinal muscles in adolescent idiopathic scoliosis. Eur Spine J. 2016;25(10):3146-53.

16. Martinez-Llorens J, Ramirez M, Colomina MJ, Bago J, Molina A, Caceres E, et al. Muscle dysfunction and exercise limitation in adolescent idiopathic scoliosis. The European respiratory journal. 2010;36(2):393-400.

17. Lin J-j, Chen W-H, Chen P-Q, Tsauo J-Y. Alteration in shoulder kinematics and associated muscle activity in people with idiopathic scoliosis. Spine. 2010;35(11):1151-7.

18. Bruyneel AV, Chavet P, Bollini G, Mesure S. Gait initiation reflects the adaptive biomechanical strategies of adolescents with idiopathic scoliosis. Annals of physical and rehabilitation medicine. 2010;53(6-7):372-86.

19. Bruyneel A-V, Chavet P, Bollini G, Allard P, Berton E, Mesure S. Dynamical asymmetries in idiopathic scoliosis during forward and lateral initiation step. Eur Spine J. 2009;18(2):188-95.

20. Daryabor A, Arazpour M, Sharifi G, Bani MA, Aboutorabi A, Golchin N. Gait and energy consumption in adolescent idiopathic scoliosis: A literature review. Annals of physical and rehabilitation medicine. 2017;60(2):107-16.

21. Lenssinck M-LB, Frijlink AC, Berger MY, Bierma-Zeinstra SM, Verkerk K, Verhagen AP. Effect of bracing and other conservative interventions in the treatment of idiopathic scoliosis in adolescents: a systematic review of clinical trials. Physical therapy. 2005;85(12):1329-39.

22. Romano M, Minozzi S, Zaina F, Saltikov JB, Chockalingam N, Kotwicki T, et al. Exercises for adolescent idiopathic scoliosis: a Cochrane systematic review. Spine. 2013;38(14):E883-E93.

23. Hawes MC. The use of exercises in the treatment of scoliosis: an evidence-based critical review of the literature. Pediatric rehabilitation. 2003;6(3-4):171-82.

24. Mordecai SC, Dabke HV. Efficacy of exercise therapy for the treatment of adolescent idiopathic scoliosis: a review of the literature. European Spine Journal. 2012;21(3):382-9.

25. Berdishevsky H, Lebel VA, Bettany-Saltikov J, Rigo M, Lebel A, Hennes A, et al. Physiotherapy scoliosis-specific exercises–a comprehensive review of seven major schools. Scoliosis and spinal disorders. 2016;11(1):20.

26. Bettany-Saltikov J, Parent E, Romano M, Villagrasa M, Negrini S. Physiotherapeutic scoliosis-specific exercises for adolescents with idiopathic scoliosis. Eur J Phys Rehabil Med. 2014;50(1):111-21.

27. Negrini S, Zaina F, Romano M, Negrini A, Parzini S. Specific exercises reduce brace prescription in adolescent idiopathic scoliosis: a prospective controlled cohort study with worst-case analysis. Journal of rehabilitation medicine. 2008;40(6):451-5.

28. Negrini S, Fusco C, Minozzi S, Atanasio S, Zaina F, Romano M. Exercises reduce the progression rate of adolescent idiopathic scoliosis: results of a comprehensive systematic review of the literature. Disability and rehabilitation. 2008;30(10):772-85.

29. Fusco C, Zaina F, Atanasio S, Romano M, Negrini A, Negrini S. Physical exercises in the treatment of adolescent idiopathic scoliosis: an updated systematic review. Physiotherapy theory and practice. 2011;27(1):80-114.

30. Kuru T, Yeldan I, Dereli EE, Ozdincler AR, Dikici F, Colak I. The efficacy of three-dimensional Schroth exercises in adolescent idiopathic scoliosis: a randomised controlled clinical trial. Clinical rehabilitation. 2016;30(2):181-90.

31. Schreiber S, Parent EC, Moez EK, Hedden DM, Hill D, Moreau MJ, et al. The effect of Schroth exercises added to the standard of care on the quality of life and muscle endurance in adolescents with idiopathic scoliosis-an assessor and statistician blinded randomized controlled trial: "SOSORT 2015 Award Winner". Scoliosis. 2015;10:24.

32. Ko K-J, Kang S-J. Effects of 12-week core stabilization exercise on the Cobb angle and lumbar muscle strength of adolescents with idiopathic scoliosis. Journal of exercise rehabilitation. 2017;13(2):244.

33. Akuthota V, Nadler SF. Core strengthening. Archives of physical medicine and rehabilitation. 2004;85:86-92.

34. Imai A, Kaneoka K, Okubo Y, Shiina I, Tatsumura M, Izumi S, et al. Trunk muscle activity during lumbar stabilization exercises on both a stable and unstable surface. Journal of orthopaedic & sports physical therapy. 2010;40(6):369-75.

35. Gür G, Ayhan C, Yakut Y. The effectiveness of core stabilization exercise in adolescent idiopathic scoliosis: A randomized controlled trial. Prosthetics and orthotics international. 2017;41(3):303-10.

36. Shin SS, Lee YW, Song CH. Effects of lumbar stabilization exercise on postural sway of patients with adolescent idiopathic scoliosis during quiet sitting. Journal of physical therapy science. 2012;24(2):211-5.

37. Reem J, Carney J, Stanley M, Cassidy J. Risser sign inter-rater and intra-rater agreement: is the Risser sign reliable? Skeletal radiology. 2009;38(4):371-5.

38. Horne JP, Flannery R, Usman S. Adolescent idiopathic scoliosis: diagnosis and management. Am Fam Physician. 2014;89(3):193-8.

39. Langensiepen S, Semler O, Sobottke R, Fricke O, Franklin J, Schonau E, et al. Measuring procedures to determine the Cobb angle in idiopathic scoliosis: a systematic review. Eur Spine J. 2013;22(11):2360-71.

40. Mannion AF, Knecht K, Balaban G, Dvorak J, Grob D. A new skin-surface device for measuring the curvature and global and segmental ranges of motion of the spine: reliability of measurements and comparison with data reviewed from the literature. European Spine Journal. 2004;13(2):122-36.

41. Livanelioglu A, Kaya F, Nabiyev V, Demirkiran G, Fırat T. The validity and reliability of “Spinal Mouse” assessment of spinal curvatures in the frontal plane in pediatric adolescent idiopathic thoraco-lumbar curves. European Spine Journal. 2016;25(2):476-82.

42. Lenke LG, Betz RR, Harms J, Bridwell KH, Clements DH, Lowe TG, et al. Adolescent idiopathic scoliosis: a new classification to determine extent of spinal arthrodesis. JBJS. 2001;83(8):1169-81.

43. Lenke LG, Edwards CC, Bridwell KH. The Lenke classification of adolescent idiopathic scoliosis: how it organizes curve patterns as a template to perform selective fusions of the spine. Spine. 2003;28(20S):S199-S207.

44. Sanders JO, Polly Jr DW, Cats-Baril W, Jones J, Lenke LG, O’Brien MF, et al. Analysis of patient and parent assessment of deformity in idiopathic scoliosis using the Walter Reed Visual Assessment Scale. Spine. 2003;28(18):2158-63.

45. Asher M, Lai SM, Burton D, Manna B. The reliability and concurrent validity of the scoliosis research society-22 patient questionnaire for idiopathic scoliosis. Spine. 2003;28(1):63-9.

46. Coelho DM, Bonagamba GH, Oliveira AS. Scoliometer measurements of patients with idiopathic scoliosis. Brazilian journal of physical therapy. 2013;17(2):179-84.
